# Adipocyte derived exosomes promote cell invasion and challenge paclitaxel efficacy in ovarian cancer

**DOI:** 10.1186/s12964-024-01806-4

**Published:** 2024-09-16

**Authors:** Michael Ellis Williams, David Howard, Claire Donnelly, Fereshteh Izadi, Jezabel Garcia Parra, Megan Pugh, Kadie Edwards, Kerryn Lutchman-Sigh, Sadie Jones, Lavinia Margarit, Lewis Francis, R. Steven Conlan, Francesca Taraballi, Deyarina Gonzalez

**Affiliations:** 1 Swansea University Medical School, Faculty of Medicine, Health and Life Science, Swansea University Singleton Park, Swansea, Wales, SA2 8PP UK; 2 Department of Gynaecology Oncology, Singleton Hospital, Swansea Bay University Health Board, Swansea, Wales, SA2 8QA UK; 3 Department of Obstetrics and Gynaecology, University Hospital of Wales, Cardiff and Vale University Health Board, Cardiff, UK; 4 Department of Obstetrics and Gynaecology, Princess of Wales Hospital, Cwm Taf Morgannwg University Health Board, Bridgend, Wales, CF31 1RQ UK; 5 Center for Musculoskeletal Regeneration, Houston Methodist Orthopedics & Sports Medicine, Houston Methodist Research Institute, Houston, TX USA

**Keywords:** Ovarian cancer, Omentum, Adipocytes, Exosomes, miRNA, Mir-21, Prolactin, Obesity

## Abstract

**Background:**

Epithelial ovarian cancer (EOC) is the deadliest gynaecological cancer with high mortality rates driven by the common development of resistance to chemotherapy. EOC frequently invades the omentum, an adipocyte-rich organ of the peritoneum and omental adipocytes have been implicated in promoting disease progression, metastasis and chemoresistance. The signalling mechanisms underpinning EOC omentum tropism have yet to be elucidated.

**Methods:**

Three-dimensional co-culture models were used to explore adipocyte-EOC interactions. The impact of adipocytes on EOC proliferation, response to therapy and invasive capacity was assessed. Primary adipocytes and omental tissue were isolated from patients with ovarian malignancies and benign ovarian neoplasms. Exosomes were isolated from omentum tissue conditioned media and the effect of omentum-derived exosomes on EOC evaluated. Exosomal microRNA (miRNA) sequencing was used to identify miRNAs abundant in omental exosomes and EOC cells were transfected with highly abundant miRNAs miR-21, let-7b, miR-16 and miR-92a.

**Results:**

We demonstrate the capacity of adipocytes to induce an invasive phenotype in EOC populations through driving epithelial-to-mesenchymal transition (EMT). Exosomes secreted by omental tissue of ovarian cancer patients, as well as patients without malignancies, induced proliferation, upregulated EMT markers and reduced response to paclitaxel therapy in EOC cell lines and HGSOC patient samples. Analysis of the omentum-derived exosomes from cancer patients revealed highly abundant miRNAs that included miR-21, let-7b, miR-16 and miR-92a that promoted cancer cell proliferation and protection from chemotherapy when transfected in ovarian cancer cells.

**Conclusions:**

These observations highlight the capacity of omental adipocytes to generate a pro-tumorigenic and chemoprotective microenvironment in ovarian cancer and other adipose-related malignancies.

**Supplementary Information:**

The online version contains supplementary material available at 10.1186/s12964-024-01806-4.

## Background

Epithelial ovarian cancer (EOC) has the highest mortality rate of any gynaecological malignancy, with 5-year survival rates of less than 50% [1, 2]. This high mortality rate can be attributed to late presentation of metastatic disease, which can disseminate extensively within the peritoneal cavity before becoming symptomatic [3]. A primary target of local metastasis is the omentum, an adipocyte-rich pad which covers the bowel and abdominal cavity and regulates aspects of peritoneal homeostasis including inflammation, angiogenesis, immune response and metabolism [4]. The omentum therefore plays an important role in generating a pro-metastatic tumour microenvironment within the peritoneal cavity [5]. Furthermore, obesity is a risk factor for both incidence and prognosis of ovarian cancer [6, 7] and can lead to dysregulation and chronic inflammation of adipose tissue [8, 9].

Alongside late disease presentation, poor patient survival-rates are also attributed to the high rates of development of ovarian cancer resistance to platinum- and taxane-based chemotherapy, with patients with Stage III or IV ovarian cancer showing relapse rates of 70–95% [10] Interactions in the omental metastatic niche can play an important role in the development of chemoresistance [8]. Adipocytes and omental-derived stromal cells can enhance chemoresistance in ovarian cancer [11–13] and targeting adipocyte-derived signalling via FABP4 knockdown can sensitize ovarian tumour cells to platinum-based therapy [14]. While the mechanisms behind adipocyte-induced chemoresistance in ovarian cancer remain unclear, evidence from a variety of carcinomas suggests a role for cancer cell “stemness”, which enables cells to undergo drastic phenotypic changes such as the epithelial-mesenchymal-transition (EMT), and therefore evade treatment. Adipocyte-derived fatty acids can induce EMT in hepatocellular carcinoma and breast cancer [15, 16], while stemness in ovarian cancer cells has been linked to fatty acid metabolism [17].

Establishment of the omental metastatic niche involves complex bidirectional signalling between cancer cells and diverse omental stroma. A role for adipocyte-secreted interleukin-6 (IL-6) and interleukin-8 (IL-8) in the migration and invasion of ovarian cancer cells to omental tissue has been established in vitro [18]. However, a more comprehensive understanding of signalling mechanisms acting across the peritoneum is required to understand ovarian cancer omentum tropism, the activation of cancer-associated stroma and the development of chemoresistance. Extracellular vesicles (EVs), particularly exosomes, 30–150 nm vesicles secreted by a diverse range of cells, have been implicated in signalling at a distance in the generation of metastatic microenvironments in a number of malignancies [19]. Exosomes carry a cargo of bioactive molecules including proteins, lipids, metabolites, RNA and DNA, and therefore have the capacity to alter cellular activity at sites distant from their cell of origin. In ovarian cancer, tumour-derived exosomes carrying MMP1 mRNA can induce destruction of peritoneal mesothelial cells in mouse via apoptotic cell death, suggesting a role for exosomes in peritoneal dissemination [20], while exosomes derived from cancer-associated adipocytes and fibroblasts can induce chemoresistance through the transfer of microRNA-21 [21]. Exosomes, therefore, represent a mechanism for bidirectional modification of a tumour and its microenvironment through the exchange of a range of bioactive molecules.

Here, we use three dimensional (3D) models to explore ovarian cancer-adipose interactions. We observe striking differences in the response of EOC cell lines to adipocyte signalling. We find adipocytes are able to induce chemoresistance and EMT in a subset of EOC cells and explore exosomes as a potential factor in adipose-tumour paracrine signalling. We find that omentum-derived exosomes, regardless of the presence of malignant disease, increase EMT marker expression in ovarian cancer cell lines and patient samples and exosomal microRNAs reduce sensitivity to paclitaxel treatment in e-cadherin^+^, pre-EMT EOC cells.

## Methods

### Patient samples and tissue-conditioned media

Omentum samples from cancer and non-cancer patients and ascites samples collected from cancer patients used in this work were collected during routine diagnostic and debulking surgery. Ethical approval was granted from HRA NHS REC Wales 6 Research Ethics Committee (LREC15/WA/0065), and written, informed consent was obtained from patients prior to enrolment into the study. Patients were recruited from Gynaecology Oncology clinics, and they were subsequently diagnosed with benign or malignant ovarian masses. Patients with infection, chronic inflammation, autoimmune disease and other cancers were excluded from this study. None of the patients in the control or study group were on exogenous hormones. Patient diagnoses and samples are detailed in Table 1. To generate omental tissue-conditioned media (OT-CM), samples were cut into approximately 1cm^3^ pieces and bathed in 2 ml primary ovarian media consisting of 1:1 mixture of MCDB: Media 199 supplemented with 20% FBS and 1% Penicillin-Streptomycin (P/S). After 24 h, media was collected and filtered through 22 μm filters to remove any cells and tissue debris. Adiponectin content of conditioned media was assessed via enzyme-linked immunoabsorbant assay (ELISA) (eBioscience, Austria). Tissue conditioned media from ovarian samples was collected as above as adiponectin^−^ control.

### Primary adipocyte isolation and culture

For primary adipocyte isolation, omental tissue was then cut into small pieces (∼ 1cm^3^) and digested with 2 mg/ml collagenase for 4 h with agitation by vortexing every 15–30 min. Adipocyte fraction was isolated centrifugation and suspended in adipocyte media consisting DMEM/F12 supplemented with 10% FBS, 1% P/S, 8 µg/ml d-biotin (Sigma-Aldrich, UK), 0.5 µg/ml insulin and 400ng/ml dexamethasone (Abcam, UK). Isolated adipocytes were seeded into 96-well plastic culture plates which had been previously incubated with 100% FBS to aid attachment. Following attachment, media was replaced, and cells grown for 24 h to generate primary adipocyte conditioned media. As above, adiponectin content of primary adipocytes was assessed via ELISA. Media was conditioned in non-adipocyte ovarian stromal cells for use as adiponectin^−^ control. Lipid content was assessed via Oil Red staining as per supplier’s instructions (Abcam, UK).

### Cell line culture

Ovarian cancer cell lines SKOV3 (CVCL_0532), OVCAR-3 (CVCL_0465), UWB1.289 (BRCA1mut; CVCL_B079), UACC1598 (CVCL_4040) and CAOV3 (CVCL_0201) were purchased from American Type Culture Collection and cultured as per supplier’s instructions. SKOV-3 cells engineered to stably express luciferase (SKOV-3/LUC)(Cambridge Bioscience, UK) were cultured as per SKOV-3 cells with the addition of 2mM L-glutamine and 1 µg/ml Puromycin to ensure maintenance of luciferase expressing phenotype.

### Adipocyte-derived stem cell culture and adipogenesis analysis

For Adipocyte differentiation, adipose-derived stem cell spheroid populations (ADSCs) (Invitrogen, Massachusetts, USA) were seeded as per supplier’s instructions and grown for 48 h in MesenPro RS Media (Gibco, Massachusetts, USA) until media was replaced with StemPro Adipogenesis Media (Gibco). Differentiation took 14 days to proceed, after which time lipid droplets were visible on differentiated adipocytes.

### Adipocyte - cancer cell co-culture and paclitaxel treatment

For transwell co-culture, ADSCs were seeded at 1.4 × 10^3^ cells/well in transwell inserts and expanded in MesenPro RS Media for 48 h prior to replacement for 14 days with StemPro Adipogenesis Media or maintenance in MesenPro RS Media for undifferentiated control. Cancer spheroids were generated in ultra-low attachment plates (Corning, New York, USA) (2 × 10^3^ cells/well) for 24 h prior to insertion of adipocyte-containing transwell inserts on top of spheroids for co-culture. Co-culture proceeded for 72 h, and end-point cell viability was measured with CellTiter-Glo Luminescent Cell Viability Assay (Promega).

For SKOV-3/LUC-Adipocyte co-culture, Adipocyte and U-ADSC spheroids were generated as described above. Following 14 days in spheroid culture, 2 × 10^3^ SKOV/LUC cells were added to spheroid culture. Co-culture proceeded for 96 h, or for drug response assays for 24 h before the addition of paclitaxel and incubation for 72 h. Cell viability was measured using Pearson Firefly Luciferase Assay (Thermo Fisher, Massachusetts, USA) as per supplier’s instructions. Reagents and cells were mixed by vigorous pipetting followed by shaking at 300 rpm at room temperature for 5 min to ensure spheroid disaggregation.

### Exosome isolation and treatment

Exosomes were isolated from 10 ml OT-CM using qEV original column, according to manufacturer’s instructions (Izon Science). Prior to sample loading, the OT-CM samples were concentrated using the Centriprep 10 K filter device (Merck Millipore) according to manufacturer’s instructions. This concentrated 10 ml of OT-CM to a final volume of 500-600 ml. After rinsing the column with PBS (0.02 μm filtered), 500 ml of the concentrated OT-CM was added to the top of the qEV column. Subsequently, EV fractions of 500 µl were collected. Purified exosomes were resuspended in 10 ml primary ovarian media, while for exosome-concentrated treatments purified exosomes were resuspended in 5 ml primary ovarian media. Exosome-depleted treatments were generated via ultracentrifugation. Particle size distribution was measured by dynamic light scattering (DLS, Malvern Zetasizer nanoseries) and nanoparticle tracking analysis (NTA) (ZetaView PMX 110 V3.0, Particle Metrix, USA).

Exosome-specific protein (CD9, CD63 and CD81) content in samples was analysed using ExoELISA-ULTRA assays (Systems Bioscience) as per supplier’s instructions. Colorimetric ELISA measurements were normalized to particle number, as measured by nanoparticle tracking analysis. For cell treatment, cell lines were seeded at 1 × 10^4^ cells/well in 96-well 350µL White Clear Bottom Plates (Porvair, UK) and treated with 200µL 1:1 primary ovarian media: treatment media. Population viability was measured using RealTime-Glo MT Assay (Promega, UK).

### EMT marker analysis

EMT marker profiles for 2-and 3D cultured SKOV-3 and OVCAR-3 populations were characterized, both in monoculture and adipocyte co-culture. For protein analysis, immunoblotting was carried using primary antibodies for E-Cadherin (clone: G10)(Santa Cruz Biotech), N-Cadherin (clone: 13A9) (Santa Cruz Biotech), and Vimentin (clone: V9) (Santa Cruz Biotech), normalized to GAPDH (clone: O411) (Santa Cruz Biotech). Differences between profiles of 2- and 3D cultured populations were determined via RT-qPCR.

For fluorescence analysis of tumor spheroids, immunostaining was carried out as described by Weiswald and colleagues [17]. Briefly, 2 × 10^3^ cells were grown as spheroids via the hanging drop method for 72 h before fixing in PBS containing 4% PFA 1% Triton for 3 hours at 4^o^c. Following washing with PBS, spheroids were dehydrated and rehydrated via sequential incubation with 25%, 50%, 75% and 95% methanol for 30 min, before 5 h in 100% methanol and reversal of the dehydration sequence to 0% methanol 100% PBS. Spheroids were blocked overnight at 4^o^c in PBST containing 3% BSA, followed by incubation with primary antibodies for E-Cadherin (clone: EP700Y) (Abcam), N-Cadherin (as above) and Vimentin (Abcam, ab24525) for 48 h and fluorophore-labelled anti-mouse (ab150117), anti-rabbit (Abcam, ab150086) and anti-chicken (Abcam, ab150175) secondary antibodies. Prior to visualization, spheroids were co-stained with Hoechst and transferred to microscope slides.

### Immunoblotting of exosome-treated spheroids

OVCAR-3 cells seeded as above (2,000 cells per well of a 96-well agarose-coated plate) and incubated at 37˚C to allow formation of spheroids. NTA analysis presented a concentration of 1.09 × 10^9^ particles per ml, used to calculate a treatment concentration of 1.4 × 10^4^ particles per cell (in 25 µl PBS treatment volume). Spheroids were treated with exosomes at 24 h and again at 48 h post-formation, before harvesting at 72 h for protein extraction (as described above). PBS used as a vehicle control. Following protein extraction, exosome-treated OVCAR-3 spheroids were compared to untreated OVCAR-3 spheroid protein samples in an immunoblot using the same antibodies as described above, for analysis of differences in E-cadherin, N-cadherin, and vimentin expression.

### Ovarian cancer patient-derived spheroids culture and treatments

Ascites samples were collected from HGSOC patients (*n* = 8), spheroids isolated and grown as 3D spheroids using 96-well ultra-low attachment plates. Spheroids were then treated with OT-CM and with omentum-derived exosomes isolated from omentum samples of patients diagnosed with HGSOC or benign conditions. After 72hs incubation, cell viability measured by CellTiter-Glo^®^ 3D Cell Viability Assay (Promega, UK) and protein extracted from spheroids interrogated for the levels of E-cadherin, N-cadherin, and vimentin expression by immunoblot (as described above). To assess the effect on response to paclitaxel treatment, spheroids were incubated with omentum-derived EVs alone or in combination with paclitaxel for 72 h, and cell viability was measured using the CellTiter-Glo^®^ 3D Cell Viability Assay (Promega, UK).

### miRNA analysis by next generation sequencing

The miRNA sequencing from purified exosome samples was performed at QIAGEN Genomic Services (Hilden, Germany). Briefly, exosomes were isolated from the OC-CM of omental biopsy samples collected from HGSOC patients (*n* = 3) or from patients diagnosed with benign masses (*n* = 3). Concentration of exosome samples was determined by NTA and RNA was isolated from 200 µl using the miRNeasy Serum/Plasma Kit (QIAGEN) according to manufacturer’s instructions with an elution volume of 14 µl. Briefly, the library preparation was done using the QIAseq miRNA Library Kit (QIAGEN). A total of 5 µl total RNA was converted into miRNA NGS libraries. After adapter ligation, UMIs were introduced in the reverse transcription step. The cDNA was amplified using PCR (22 cycles) and during the PCR indices were added. After PCR the samples were purified. Library preparation was quality controlled using capillary electrophoresis (Agilent DNA 1000 Chip). Based on quality of the inserts and the concentration measurements the libraries were pooled in equimolar ratios. The library pool(s) were quantified using qPCR. The library pool(s) were then sequenced on a NextSeq (Illumina Inc.) sequencing instrument according to the manufacturer instructions (1 × 75, 1 × 8). The raw read count data was received from Qiagen. The raw read count data was generated from mapping the short reads to miRBase version 22. Briefly, the reads are processed by trimming of the common sequence, (Unique Molecular Identifier UMI) and adapters, and filtering of reads with length < 15 nucleotides or length > 55 nucleotides. They are then deduplicated using their UMI. Reads are grouped into UMI groups when they start at the same position based on the end of the read to which the UMI is ligated. Groups that contain only one read (singletons) are merged into non-singleton groups if the singleton’s UMI can be converted to a UMI of a non-singleton group by introducing an SNP (the biggest group is chosen).

### Pathway analysis and ontology

Differential expression analysis aims at selecting miRNAs with similar expression profile between cancer and benign groups was performed using DESeq2 R package (version 1.34.0) with P-value > 1 & absolute log_2_ fold change < 1). Heatmap of selected miRNAs between the two groups was then created using VST transformed values (scaled) using heatmap R package (version 1.0.12). For pathway analysis, we firstly identified experimentally validated target genes (functional validation) of each of selected miRNAs from miRTarBase (release 9.0) and then performed pathway analysis of these target genes using KEGG 2021 HUMAN and DisGeNET (https://www.disgenet.org/) databases and clusterProfiler package was to use to visualize the data. The biological pathways with an adjusted p-value < 0.05 were considered statistically significant.

### Transfection of miRNA

To explore whether adipocyte derived exosomal miRNA affects ovarian cancer cell proliferation and response to treatment, we performed miRNA transfection. OVCAR-3 cells seeded 1 × 10^3^ cells per well of a 96-well plate and incubated overnight at 37˚C, or until 80–90% confluent. The next day, cells were transfected with 5nM miScript miRNA mimics of let-7b-5p, miR-16-5p, miR-21-5p and miR-92a-3p (Qiagen), or a non-targeting siRNA cocktail (Horizon Discovery, UK, Cat. No. D-001810-10-05) using HiPerFect Transfection Reagent (Qiagen) according to the manufacturer’s fast-forward protocol. Transfected cells were treated with paclitaxel 5nM, or vehicle control for 48 h and viability measured using the RealTime-Glo MT Assay.

### Statistical analysis

Statistical analysis was performed using GraphPad Prism 8 for Windows (GraphPad Sofware). Results are reported as mean ± SD from at least three independent replicates unless otherwise stated, with *p* ≤ 0.05 used as a threshold for significance. Differences in population viability between treatment groups was assessed via One-way ANOVA with Tukey’s Multiple Comparisons Test or Student’s t-test as indicated. Differences in gene expression between cell lines and between culture systems were assessed via Student’s t-test.

## Results

### Omental cell-secreted factors and adipocyte signalling affect ovarian cancer cell viability

**Fig. 1:**
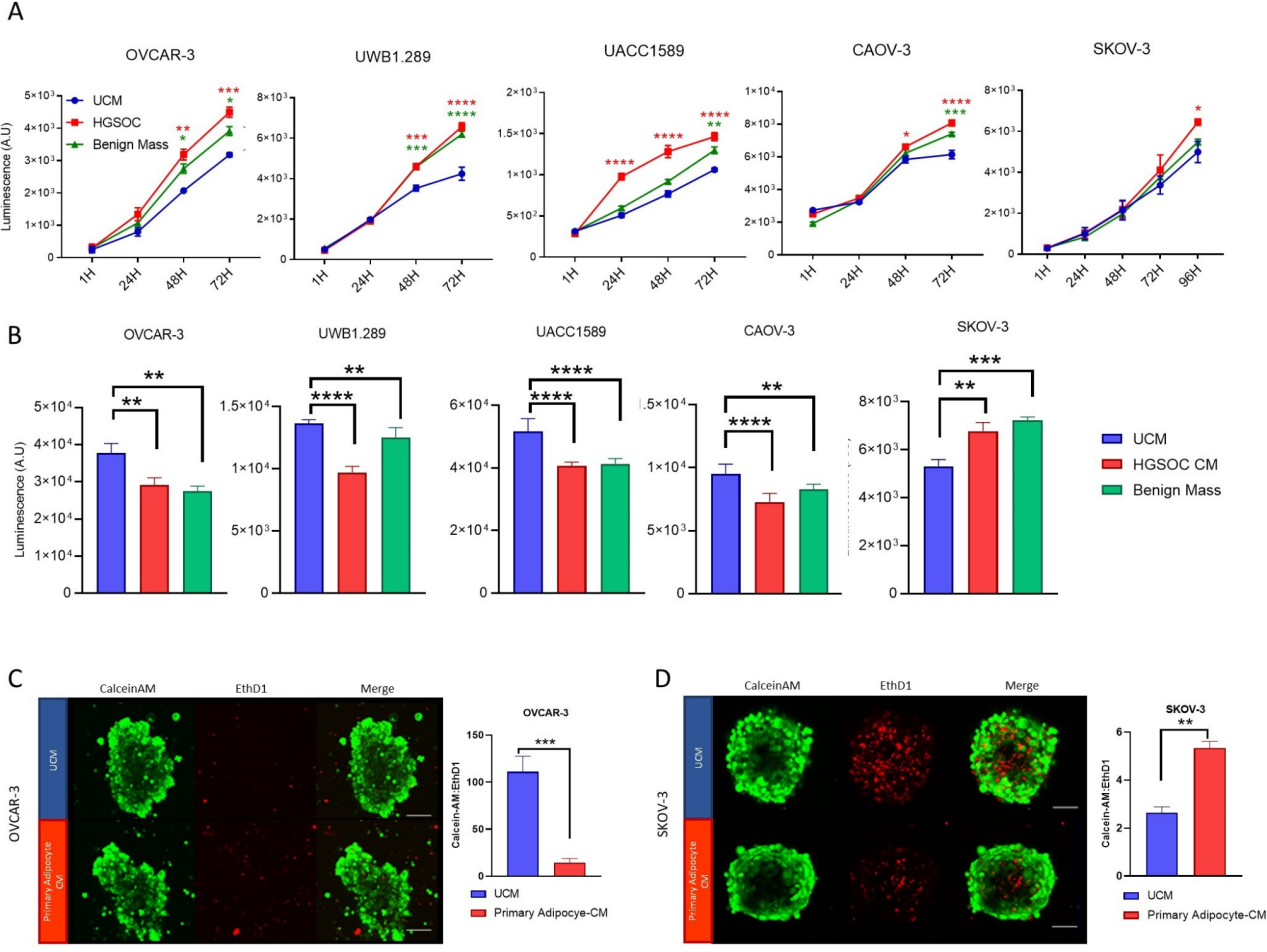
Omental secreted factors influence EOC proliferation in monolayers and spheroid populations. Ovarian cancer cell lines grown as monolayers (**A**) or 3D spheroid (**B**) were treated with unconditioned media (control), or media conditioned by patient omentum samples and cell viability was measured by Realtime-Glo (**A**) and 3D Glo (**B**) cell viability assays. Tumour spheroids generated from OVCAR-3 (**C**) and SKOV-3 (**D**) were treated with media conditioned by primary adipocytes derived from patient omentum samples and incubated with viability stains. Ratio of CalceinAM (live cells) to Ethidium homodimer (dead cells) was calculated as a measure of population viability. Data points show mean + SD, *n* ≥ 3. *p < 0.05, **p < 0.01, ***p < 0.005, ****p < 0.0005 relative to unconditioned media (UCM) control for **A** and **B** (One-way ANOVA with Tukey’s Multiple Comparisons Test) and as indicated for **C** and **D** (Student’s t-test). Scale bar = 100 μm

Metastasizing ovarian cancer has been shown to home to the omentum, and this organ is commonly invaded [18]. Initially, therefore, the effect of omentum-secreted factors on ovarian cancer cell lines was explored. Omental tissue was collected from two patients undergoing surgery whose diagnoses were high-grade serous ovarian carcinoma (HGSOC) and a benign ovarian mass (Table 1. Patients B, C). Tissue was used to generate omental tissue-conditioned media (OT-CM) containing tissue secretome. Adipocyte activity in these tissues was confirmed by adiponectin ELISA (Figure S1A) and ovarian cancer cell lines (SKOV-3, OVCAR-3, UACC1598, UWB1.281, CAOV3) were treated with 50% OT-CM in two-dimensional (2D) monolayer (Fig. 1A) and three-dimensional (3D) spheroid culture (Fig. 1B). An increase in proliferation was observed after 72 h in 2D monolayer cultures of OVCAR-3, UACC1598, UWB1.281 and CAOV3 and after 96 h in SKOV-3 cells treated with HGSOC OT-CM. However, striking differences in the response of the epithelial ovarian cancer (EOC) cell lines was observed in spheroid cultures. Viability was increased in SKOV-3 spheroids by treatment with OT-CM but was significantly reduced in OVCAR-3, UACC1598, UWB1.281 and CAOV3 spheroids relative to unconditioned media (UCM) control. Interestingly, similar results were obtained regardless of whether the omental sample originated from a malignant or benign ovarian mass.

**Table 1:** List of patient samples used in this study. HGSOC – high grade serous ovarian carcinoma

Patient ID	Tissue Samples	Diagnosis
Patient A	Left Ovary	HGSOC Stage 3
	Omentum	
Patient B	Left ovary	HGSOC
	Omentum	
Patient C	Left ovary	Ovarian Mass
	Omentum	
Patient D	Omentum	Benign Mass
Patient E	Omentum	Benign Mass
Patient F	Omentum	Benign Mass
Patient G	Omentum	Ovarian Cancer
Patient H	Omentum	Ovarian Cancer
Patient I	Omentum	Ovarian Cancer
Patient J	Omentum	Ovarian Cancer
Patient K	Omentum	Ovarian Cancer

To confirm the contribution of omental adipocytes to observed changes in ovarian cancer cell viability, mature adipocytes were isolated from omental tissue (Table 1, Patient A) and the effect of adipocyte-secreted factors assessed. The presence of mature adipocytes in culture was confirmed by Oil Red staining and ELISA, with non-adipocyte omental stromal cells as negative control (Figure S1B), and adipocyte-conditioned media was collected. The effect of primary adipocyte-conditioned media on viability of EOC spheroids (SKOV-3 and OVCAR-3) was assessed by fluorescent staining of conditioned media-treated cell populations with Calcein-AM and Ethidium Homodimer-1 (EthD1) to identify live and dead cells respectively in OVCAR-3 (Fig. 1C) and SKOV-3 (Fig. 1D). As seen in omental tissue CM-treated SKOV-3 spheroids, adipocyte-conditioned media was found to significantly increase SKOV-3 viability, while population viability decreased in OVCAR-3 spheroids, as quantified by the ratio of Calcein-AM^+^:Ethd1^+^ cells.

**Fig. 2:**
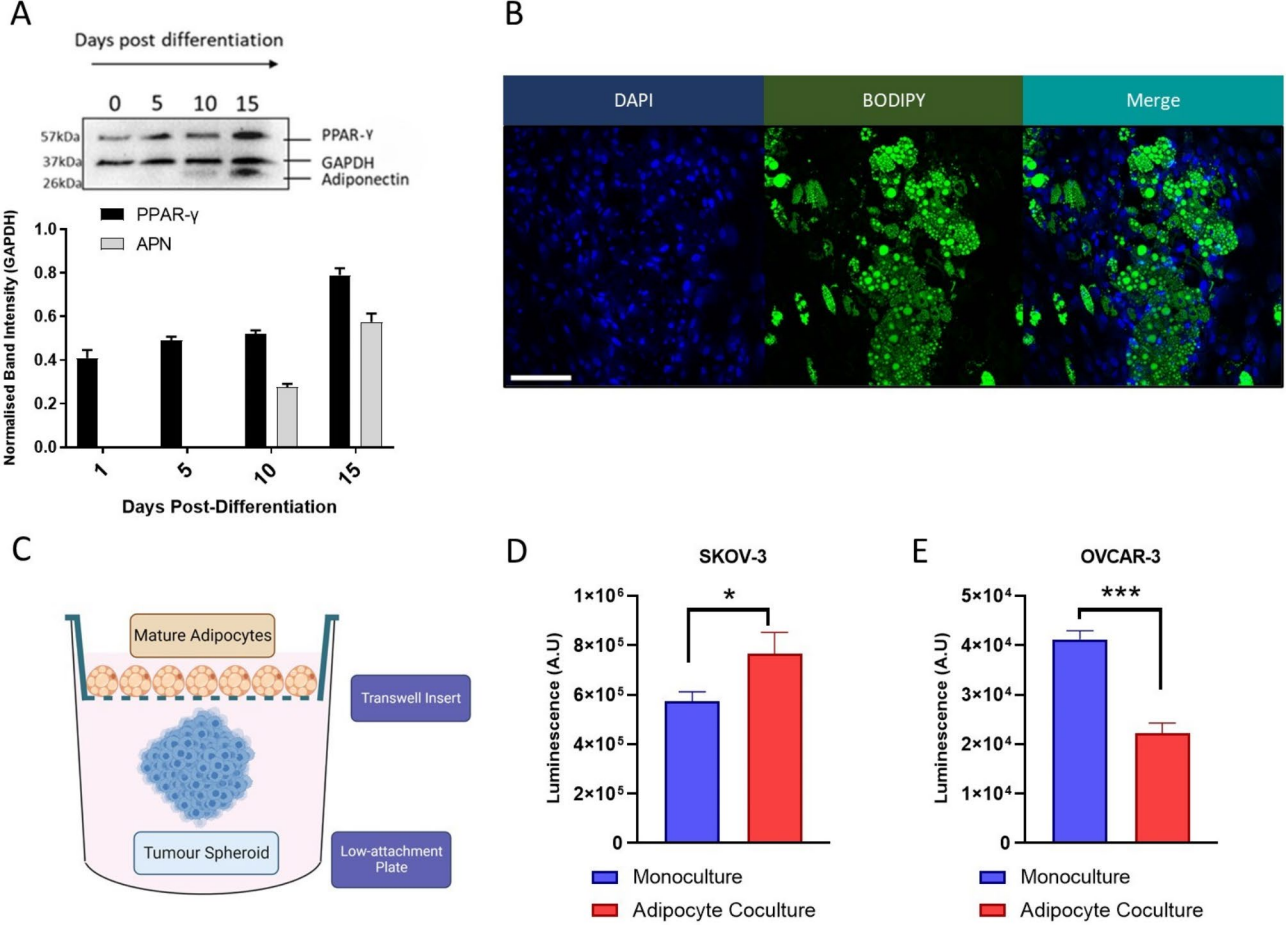
Co-culture of EOC spheroids with mature adipocytes impacts viability. Adipose-derived stem cells were differentiated into mature adipocytes and demonstrated increased expression of adipocytes specific markers PPAR-γ and Adiponectin (**A**) and developed lipid droplets, stained by neutral lipid maker BODIPY in (**B**). EOC spheroids were co-cultured alongside mature adipocytes in a transwell co-culture model displayed in diagram (**C**). Following 72 h in co-culture, population viability of spheroids was measured via Celltiter-Glo 3D viability assay for SKOV-3 (**D**) and OVCAR-3 (**E**). *N* = 3, bar plots show mean + SD, *n* ≥ 3. *p < 0.05, ***p < 0.005, (Student’s t-test)

To explore the effect of bidirectional signalling with adipocytes on ovarian cancer cell proliferation, ovarian cancer cell lines were co-cultured with mature adipocytes derived from adipose-derived stem cells (ADSCs). Differentiation of adipocytes was confirmed by the expression of adipocyte specific markers PPAR-γ and Adiponectin (Fig. 2A) via BODIPY lipid staining (Fig. 2B). Transwell co-cultures were established incorporating mature adipocytes derived from ADSCs seeded onto transwell inserts alongside SKOV-3 and OVCAR-3 spheroids (Fig. 2C). Spheroid viability after 72 h of co-culture was assessed via Celltiter-Glo 3D viability assay (Fig. 2D, E). Again, SKOV-3 (Fig. 2D) and OVCAR-3 (Fig. 2E) cell lines demonstrated striking differences in their response to adipocyte co-culture, with significant increase and decrease in population viability respectively. As ADSC-derived adipocytes constitute a heterogenous population of cells including partially differentiated or undifferentiated stem cells, the effect of undifferentiated ADSC populations on EOC growth was assessed (Figure S2). Undifferentiated ADSCs were found to have no impact on EOC spheroid viability.

An alternative co-culture system was established to assess the impact of direct contact with adipocytes on EOC growth (Fig. S3A). Here, spheroids comprising ADSC-derived mature adipocytes were established (Fig. S3B) and SKOV-3 cells stably expressing luciferase (SKOV-3/LUC) were added directly in co-culture. Luciferase activity was only detected in SKOV-3/LUC-containing cultures (Fig. S3C) and luciferase activity correlated with cell number (Fig. S3D). Strikingly, SKOV-3/LUC cells cultured directly alongside adipocytes demonstrated a 4-fold increase in luciferase activity (Fig. S3E), suggesting a significant impact on cancer cell proliferation.

### Characterization of EMT marker expression in 2D and 3D ovarian cancer cell populations

**Fig. 3:**
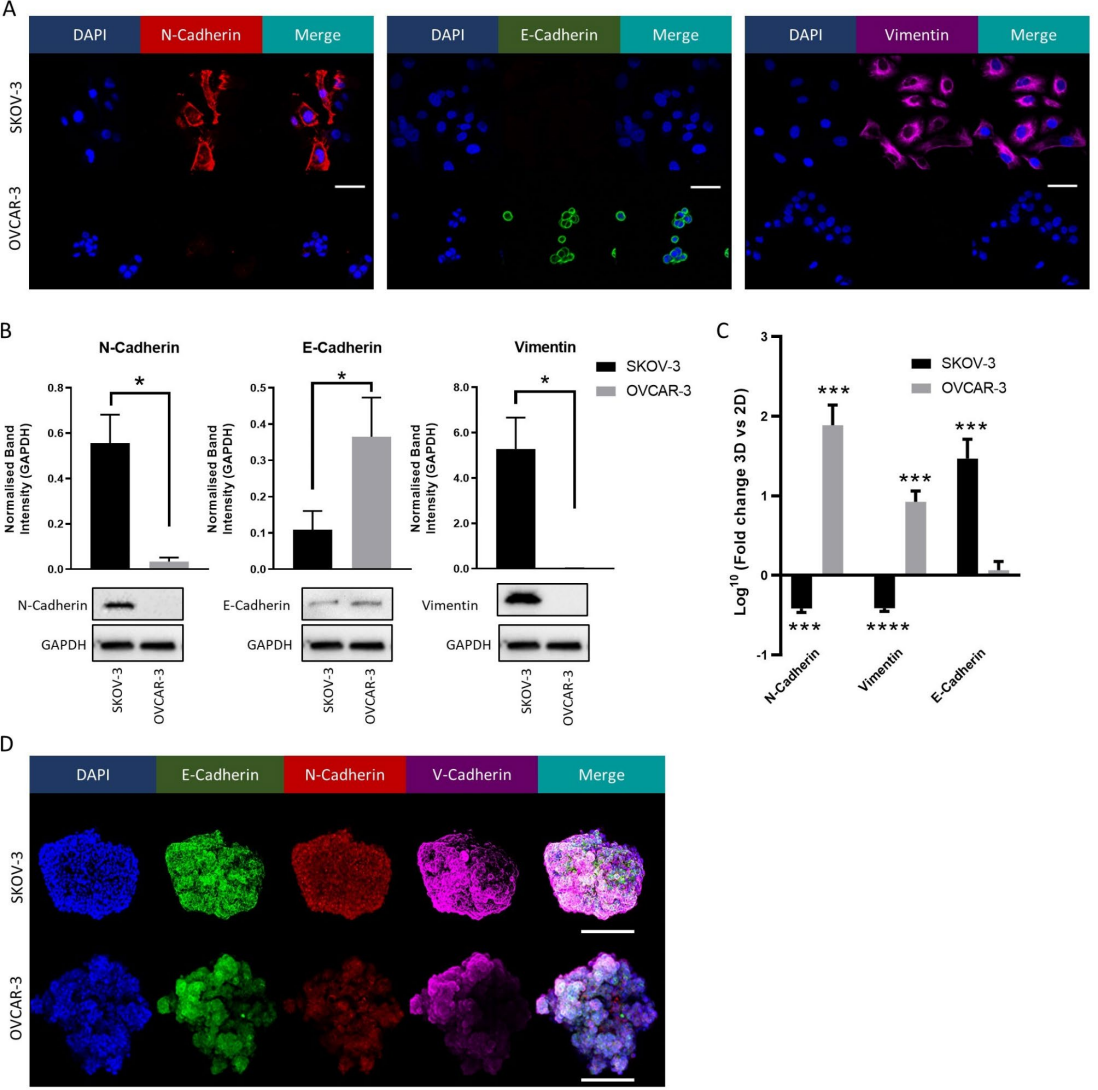
Characterisation of EMT Markers in Ovarian Cancer Cell Lines. EMT marker expression was characterized by via immunofluorescent confocal microscopy (**A**). Representative image of immunoblot and normalised quantification for EMT marker expression in EOC cell lines are given in (**B**). Changes in EMT marker profiles for cell populations grown in spheroid culture were analysed via RT-qPCR (**C**). Fold change in mRNA level (normalised to RPL-19) in 3D populations relative to 2D monolayer is displayed. Expression of EMT markers in spheroid populations was further characterized by immunofluorescent confocal microscopy (**D**). Bar plots show mean + SD, *n* = 3. *p < 0.05, **p < 0.01, ***p < 0.005, ****p < 0.0005 as indicated, or relative to 2D monolayer for **C** (unpaired Student’s T-test)

Given the observed differences in the response of epithelial ovarian cancer (EOC) cell lines to adipocyte signalling and co-culture, we explored potential sources of a differential response using SKOV-3 and OVCAR-3 cells. In particular we focus on whether changes in expression of epithelial-to-mesenchymal transition (EMT) markers in these cells are mediated by adipocyte signalling. To further explore a potential effect of adipocytes on OC EMT, initially the expression of mesenchymal cell surface markers N-Cadherin and Vimentin, along with the epithelial marker E-Cadherin, in EOC cell lines was profiled. Immunofluorescence (Fig. 3A) and immunoblotting (Fig. 3B) suggested a Vimentin^+^ N-Cadherin^+^ E-Cadherin^low^ phenotype for SKOV-3 and, conversely, a Vimentin^−^ N-Cadherin^−^ E-Cadherin^high^ profile for OVCAR-3. These data indicate an epithelial phenotype for OVCAR-3, while SKOV-3 present a mesenchymal-like, post-EMT surface marker profile associated with a more invasive pathology.

Differences were observed in the response of OVCAR-3 cells to omental signalling when grown in 2D monolayer compared to 3D spheroid (Fig. 1A, B). This was explored through RT-qPCR (Fig. 3C). OVCAR-3 spheroids displayed a many-fold increase in Vimentin and N-Cadherin mRNA respectively compared to 2D populations, while SKOV-3 spheroid populations displayed a decrease in these mesenchymal markers, and an increase in epithelial E-Cadherin (Fig. 3C). Similarly, immunofluorescent staining of spheroids indicted the presence of E-Cadherin^+^, N-Cadherin^+^ and Vimentin^+^ cells in both cell lines (Fig. 3D) These changes in EMT marker profile in cell populations grown in spheroid culture suggest differences in epithelial and post-EMT phenotypes and provide a potential explanation for differential response to adipocyte signalling.

### Adipocyte co-culture induces EMT in epithelial ovarian cancer spheroids

**Fig. 4:**
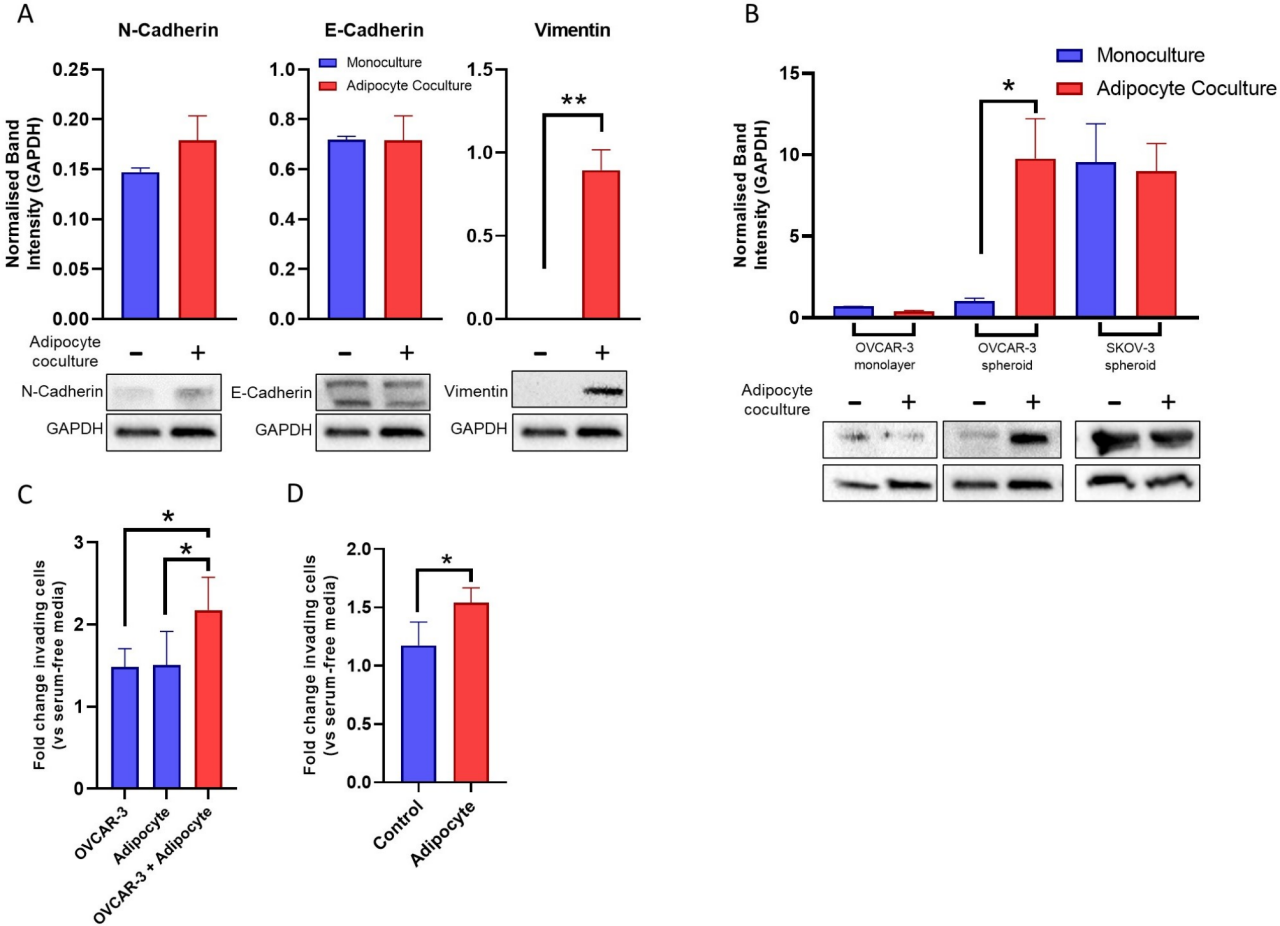
Adipocyte Co-Culture Induces EMT in Epithelial Ovarian Cancer Spheroids. EMT marker expression in OVCAR-3 populations in co-culture and mono-culture control was analysed via immunoblotting (**A**). Vimentin expression in ovarian cancer cell line populations (2D monolayer and 3D spheroid) was further characterised and quantified (**B**). The capacity of OVCAR-3 cells for invasion was assessed via ECMatrix assay with mono- and co-culture derived conditioned media in lower chamber **(C)** and in the presence of mature adipocytes in the lower chamber **(D)**. In (**D**), control value was derived from adipocyte media only in the absence of mature adipocytes. Number of invading cells relative to serum-free media is displayed. Bar plots show mean + SD, *n* = 3. *p < 0.05 as indicated (unpaired Student’s T-test)

To test the hypothesis that OVCAR-3 spheroid response to adipocyte signalling was mediated by EMT, changes in EMT marker expression was assessed following adipocyte transwell co-culture. While no significant difference in N- and E-Cadherin was detected, Vimentin expression was apparent in OVCAR-3 cells in adipocyte co-cultures, suggesting a switch to a more mesenchymal phenotype (Fig. 4A) driven by adipocyte signalling. Changes in Vimentin expression upon transwell co-culture with adipocytes were therefore explored in OVCAR-3 monolayer and spheroid cultures, alongside SKOV-3 spheroids as a positive control (Fig. 4B). Increased Vimentin expression was only apparent in OVCAR-3 spheroids, with no change detected in SKOV-3. EMT has been highlighted as an important process in cancer metastasis as such changes in phenotype are required to enable cell motility and increase invasive capacity. As such, the impact of adipocytes on invasiveness of OVCAR-3 cells was assessed. Invasion assays incorporating mono-and co-culture derived conditioned media into the lower well revealed increased invasion in OVCAR-3 cell populations in the presence of co-culture derived CM relative to both OVCAR-3 and adipocyte monoculture CM alone (Fig. 4C). Similarly, invasion assays incorporating mature adipocytes into the lower chamber revealed increased numbers of invading cells (Fig. 4D).

### Adipocytes enhance ovarian cancer paclitaxel resistance

**Fig. 5:**
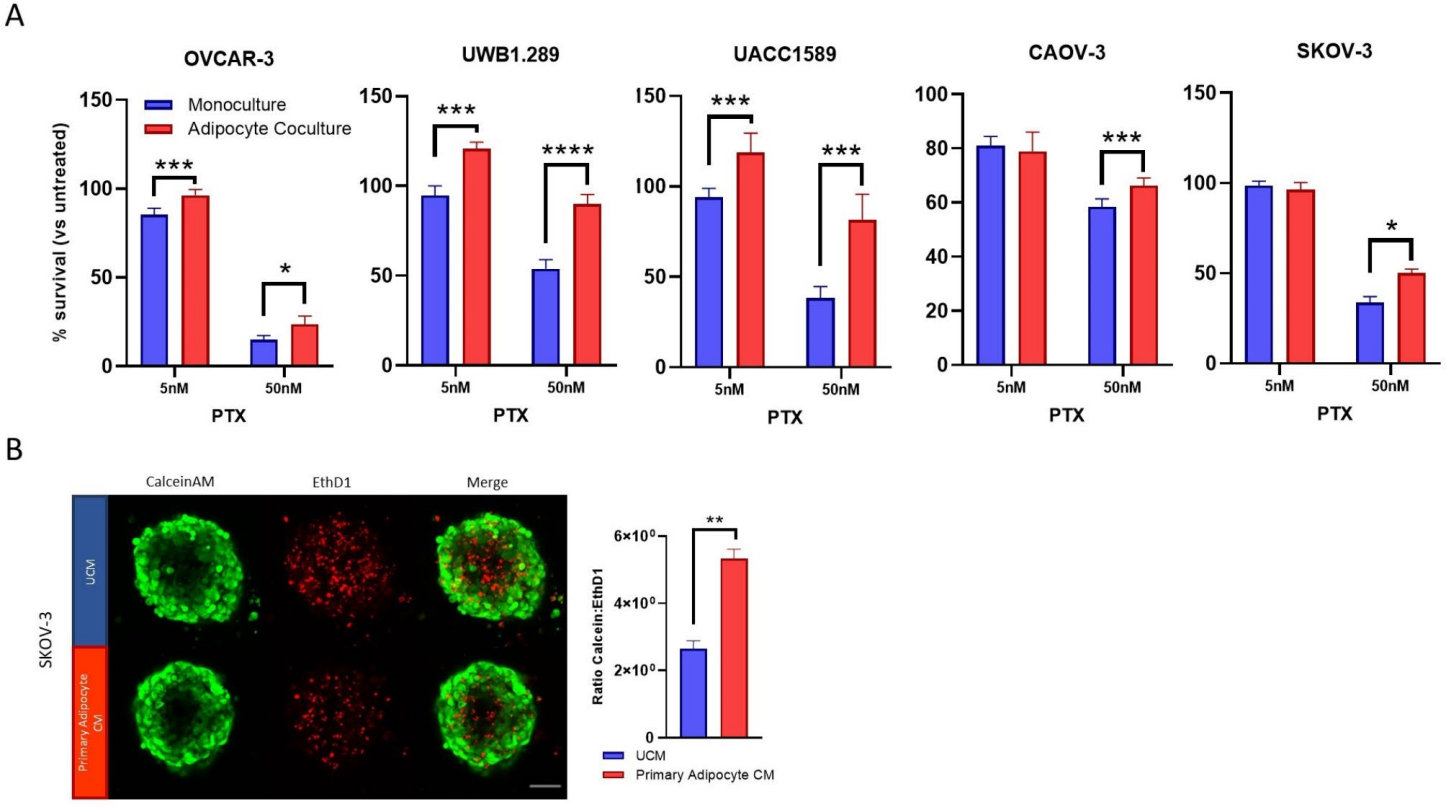
Adipocytes reduce response to paclitaxel therapy in EOC Cell Lines. (**A**) The effect of 72 h paclitaxel (PTX) treatment on ovarian cancer cell line spheroids in adipocyte co-cultures was measured using CellTiter-Glo Luminescent Cell Viability Assay and change in cell viability vs. control (no treatment) was calculated. Live/dead staining of spheroids treated with primary adipocyte media was performed with Calcein AM (green) and EthD1 (red) (**B**). The ratio of Calcein: EthD1 was calculated. Representative image displays effect of 50nM PTX treatment on SKOV-3 spheroids. Bar plots show mean + SD, *n* = 3. *p < 0.05, **p < 0.01, ***p < 0.005 as indicated (Student’s t-test)

The development of chemoresistance is a major hurdle in the effective treatment of late-stage ovarian cancer and has been associated with EMT [22]. To explore the effect of adipocyte interactions on ovarian cancer chemoresistance, all the cancer cell lines were grown in co-culture with adipocytes and were treated with taxane-based therapeutic paclitaxel (PTX). Cancer spheroids were co-cultured alongside mature adipocytes in transwell co-culture and treated with PTX in concentrations as indicated for 72 h (Fig. 5A). A significant difference was observed in the change in spheroid viability relative to untreated spheroids (% survival) in all cell lines when treated with PTX, with the exception of 5nM treatment of SKOV-3 spheroids, which did not have a significant cytotoxic effect on any group, and 5nM treatment of CAOV-3. To ensure the cytotoxic effect of PTX on adipocytes in co-culture was not having a significant impact on EOC viability indirectly, mature adipocytes were treated with 500nM PTX for 72 h and no cytotoxic effect was found (Figure S4). Furthermore, SKOV-3 spheroids grown in primary adipocyte-conditioned media exhibited reduced cell death when treated with 50nM PTX compared to those grown in unconditioned media (Fig. 5B). SKOV-3/LUC cells grown in direct contact with adipocytes were also protected against PTX treatment (Figure S3E, F).

### Omentum-derived exosomes drive ovarian cancer cell proliferation, chemoresistance and invasion via EMT

**Fig. 6:**
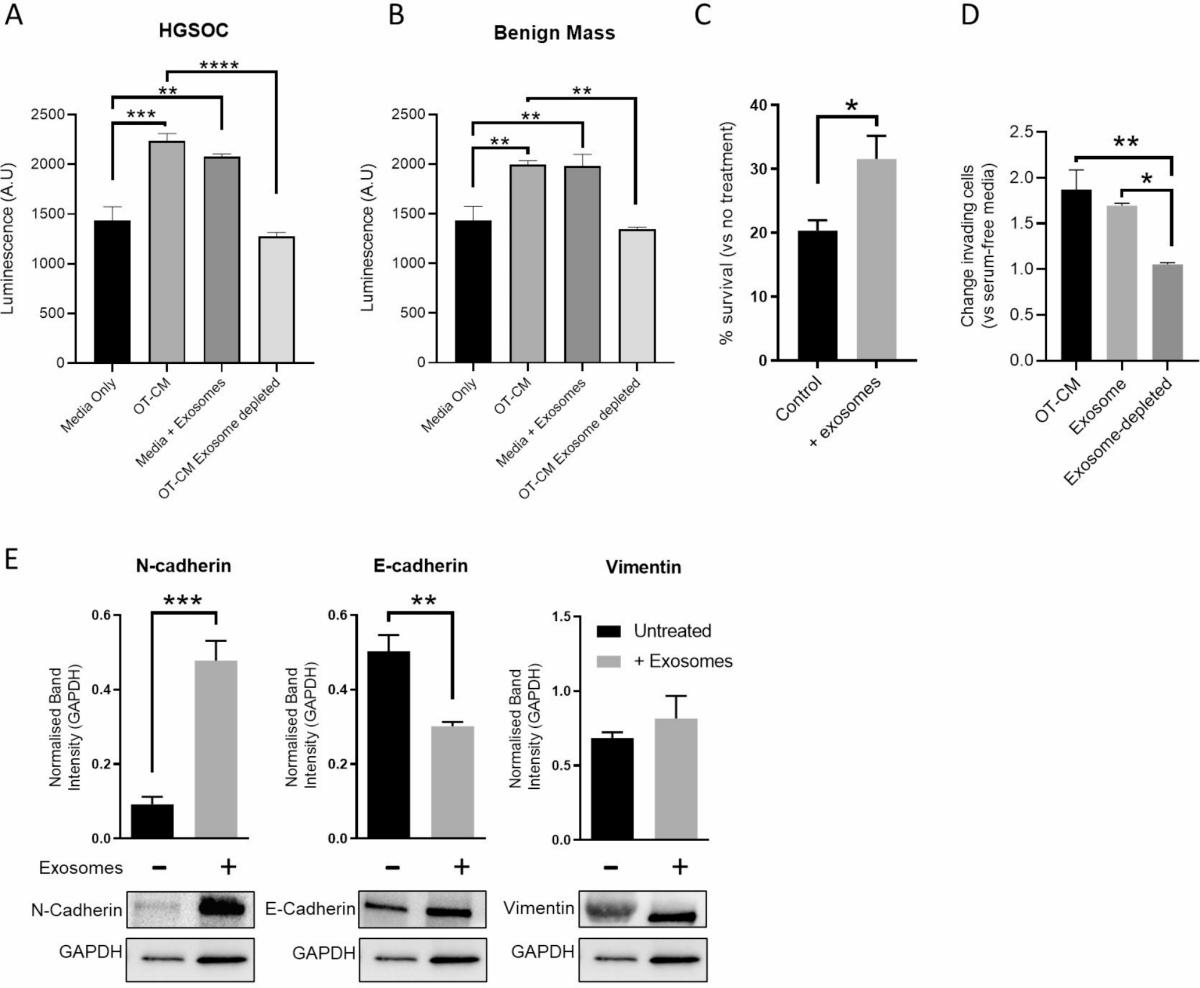
Omental tissue-derived exosomes enhance EOC proliferation, chemoprotection and invasion. Effect of EVs treatments on proliferation of OVCAR-3. EVs isolated from OT-CM derived from patients with HGSOC (**A**) and ovarian mass (**B**). Change in OVCAR-3 population viability following 72 h 50nM paclitaxel treatment was also assessed **(C).** The impact of OT-CM and OT-CM-derived EVs, as well as EV-depleted OT-CM, on OVCAR-3 invasion was assessed via ECMatrix invasion assay (**D**). Number of invading cells relative to serum-free media are displayed. EMT marker expression in OVCAR-3 spheroid populations in the presence of OT-CM-derived EVs was analysed via immunoblotting **(E**, representative images and quantification normalised to GAPDH**)**. Mean + SD, *n* ≥ 3. *p < 0.05, **p < 0.01, ***p < 0.005, ****p < 0.0005 as indicated (One-way ANOVA with Tukey’s Multiple Comparisons Test **(A**,** B**,**D)** or unpaired Student’s T-test *(C,** E)**)

Exosomes have been implicated in tumour-stroma interactions in several malignancies, including ovarian cancer [19]. A role for exosomes in omental tissue-induced ovarian cancer cell proliferation was therefore tested. Exosomes were extracted from OT-CM using qEV original columns, while OT-CM was exosome depleted by ultracentrifugation. The range of particle size present in these treatments was determined by dynamic light scattering (Fig. S5A) and the presence of exosome-specific markers CD9, CD63 and CD81 was confirmed (Fig. S5B). Treatment of OVCAR-3, UACC1598, UWB1.289 and CAOV3 cells with 6 × 10^4^ exosomes per cell for 72 h increased cell proliferation in line with the effect observed from whole OT-CM, while depletion of exosomes from OT-CM reversed pro-cancer effects of omental-derived signalling (Fig. 6A, B, Fig. S6A, B). Moreover, in cancer spheroids, treatment with omentum-derived exosomes decreased sensitivity to PTX therapy, with spheroid populations displaying significantly less change in viability under PTX treatment relative to control populations (Fig. 6C, S6C). Taken together these data suggest an important role for exosomes in adipocyte-induced changes in epithelial ovarian cancer cell lines activity.

We further assessed the role of exosomes in adipocyte-ovarian cancer interactions by exploring the impact of EVs on OVCAR-3 EMT and invasion. Depletion of exosomes from OT-CM decreased the number of invading cells in ECMatrix assay (Fig. 6D), while exosome treatment of OVCAR-3 spheroids increased the expression of the EMT marker N-cadherin while decreasing expression of epithelial marker E-cadherin (Fig. 6E). Notably, there was no significant change in Vimentin, indicating this change may be the result of alternative pathways to those observed in co-culture experiments (Fig. 4A, B).

### Omentum-derived exosomes promote proliferation of HGSOC patient-derived spheroids, chemoresistance and EMT

**Fig. 7:**
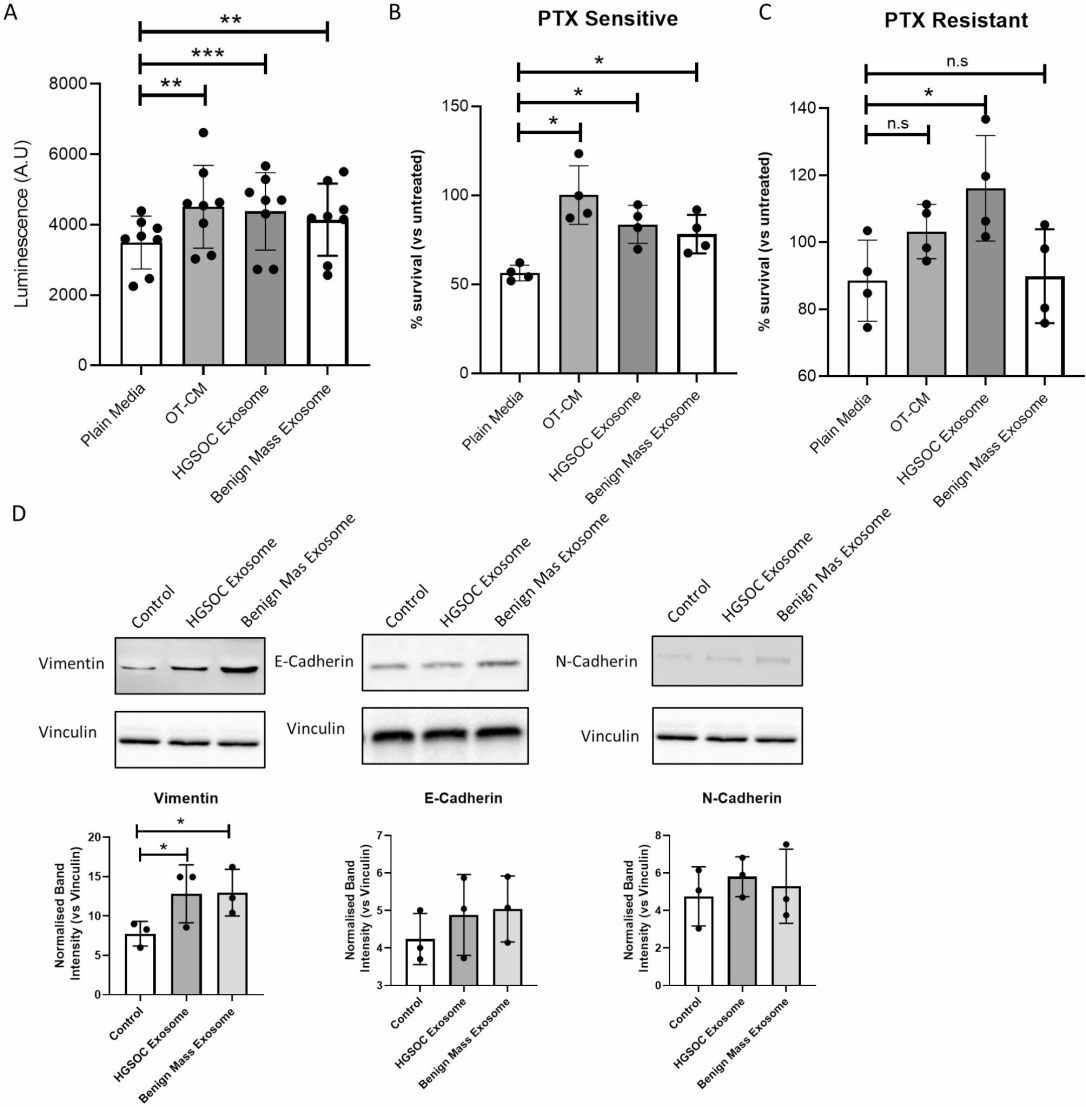
Omental tissue-derived exosomes enhance proliferation and protect from paclitaxel therapy in patient-derived cells. Effect of omental exosomes from patients with HGSOC and non-malignant ovarian mass on patient-derived ovarian cancer cells was assessed via CellTiter-Glo Luminescent Cell Viability Assay (**A**) (*n* = 8). Primary patient samples were classified based on response to paclitaxel: therapy those showing at least a 50% reduction in cell viability were labelled as sensitive, while those showing a 25% reduction or less were labelled as resistant. The impact of omental exosomes on response to paclitaxel therapy in sensitive (**B**) and resistant (**C**) primary ovarian cancer cells was assessed (*n* = 4). Expression of EMT markers Vimentin, E-Cadherin and N-Cadherin in patient samples was investigated by immunoblotting, (**D**) shows representative blot and quantified band intensity normalised to vinculin. Mean +/- SD. *p < 0.05, **p < 0.01 (One-way ANOVA with matched samples Tukey’s Multiple Comparisons test

We subsequently use spheroids derived from the ascites of HGSOC patients to validate our observations by examining the effects of OT-CM and omentum-derived exosomes on cancer cell proliferation, response to paclitaxel, and the expression of EMT markers in vitro (Fig. 7). Patient samples were divided into two groups based on their response to paclitaxel: those showing at least a 50% reduction in cell viability were labelled as sensitive, while those showing a 25% reduction or less were labelled as resistant. A statistically significant increase in ovarian cancer cell proliferation was observed in all samples after 72 h-incubation of patient-derived spheroids with OT-CM, HGSOC-omemtum exosomes or benign-omentum exosomes compared to untreated samples (Fig. 7A). We next examine the protective effect of exosomes and OT-CM to 72 h paclitaxel treatment. A significant reduction on sensitivity to paclitaxel was observed when paclitaxel sensitive patients-derived spheroids were incubated with OT-CM, as well as HGSOC omentum-exosomes and benign omentum-exosomes (Fig. 7B). However, this effect was only observed for HGSOC omentum-exosomes treatment of paclitaxel-resistant patient-derived spheroids, where the exosomes significantly increased the proliferation of cancer cells. (Fig. 7C). Finally, changes on EMT markers were identified as result of omentum-exosome treatments in these patient groups. As shown in Fig. 7D, no significant changes in E-cadherin or N-cadherin were observed after treatment of the patient-derived spheroids with omentum-exosomes but significant increases in the levels of vimentin were observed after treatment with either HGSOC or benign omentun-exosomes (Fig. 7D). This data indicates that the omentum, particularly the exosomes it secretes, mediates cell communication signals to ovarian cancer cells, thereby modifying pathways involved in cancer cell proliferation, treatment response, and EMT changes, ultimately leading to aggressive ovarian cancer phenotypes.

### Exosome-derived microRNAs increase proliferation and chemoresistance in EOC

**Fig. 8:**
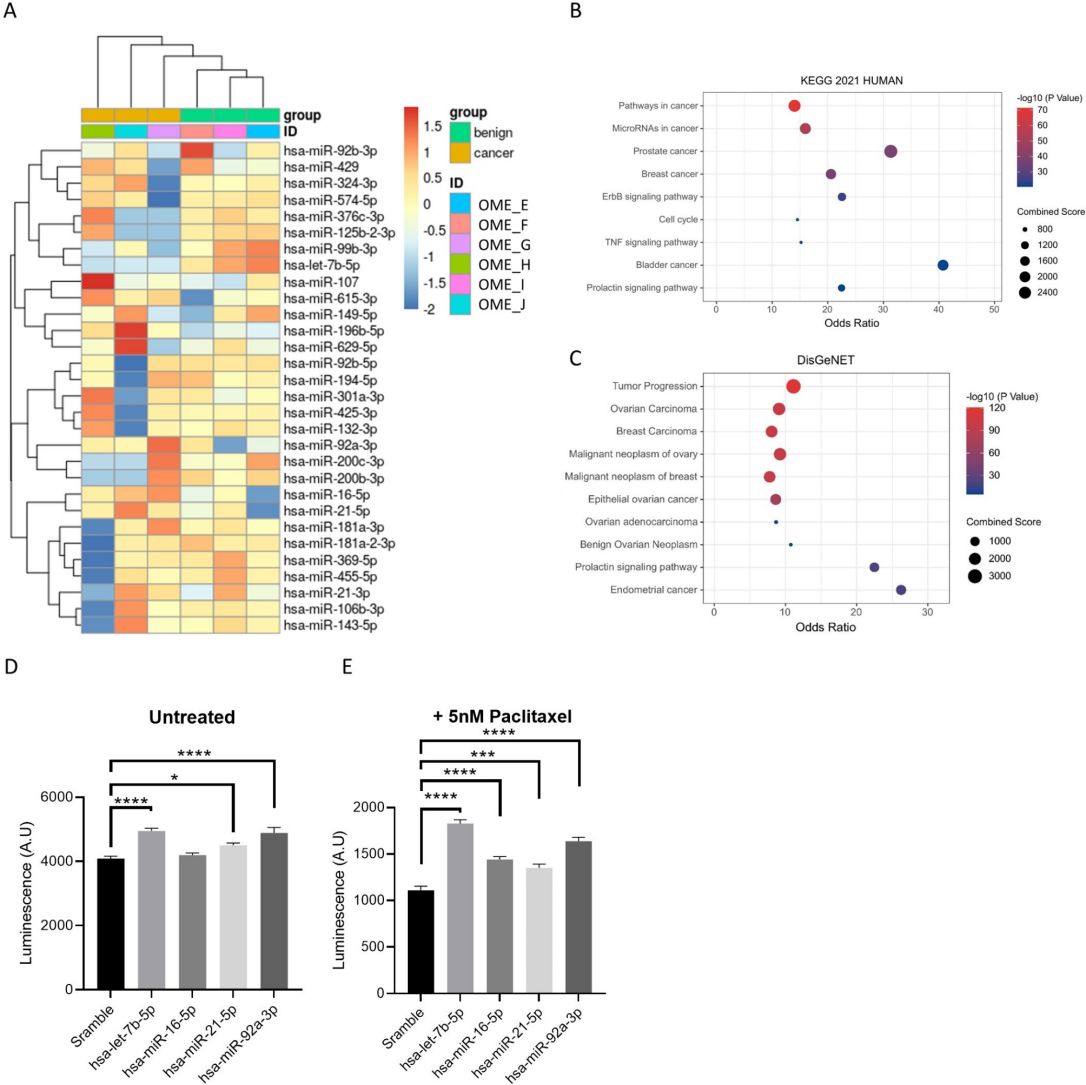
Omental exosome-expressed miRNAs induce proliferation and protect against paclitaxel therapy in EOC cells. Next generation miRNA sequencing revealed 16 miRNAs abundant in malignant and benign patient omentum samples (**A**). Pathway analysis revealed disease ontology relationships for ovarian cancer-associated pathways (**B** and **C**). The impact of transfection of miRNAs, highly expressed in omentum-derived EVS, on OVCAR-3 population viability was assessed via Realtime-Glo Cell Viability Assay **(D)**. The impact of 72 h 5nM paclitaxel treatment on miRNA transfected cells was also assessed **(E)**. Survival relative to matched untreated transfected cells is displayed. Mean + SD, *n* ≥ 5. *p < 0.05, **p < 0.01, ***p < 0.005, ****p < 0.0005 as indicated (One-way ANOVA with Tukey’s Multiple Comparisons Test)

Exosome-derived microRNAs (miRNAs) have been shown to play a substantial role in exosome signalling at a distance in various cancers, including EOC [23]. As a pro-cancer effect had been demonstrated by omental secreted factors regardless of the presence of malignancy (Figs. 1B and C, 6A and B and 7), exosomes were isolated from OT-CM of patients with both benign ovarian neoplasms and malignant tumours (Table 1, Patients D-K). miRNA next generation sequencing revealed 30 miRNAs highly abundant in exosomes derived from omental tissues (Fig. 8A). Pathway analysis of target genes using KEGG 2021 HUMAN and DisGeNET revealed top pathways including cancer and EOC-related regulation, as well as prolactin signalling pathways (Fig. 8B, C). To validate the significance of exosomal miRNAs in EOC progression and decreased response to PTX treatment, 4 miRNAs abundant in exosomes isolated from the omental secretome (let-7b, miR-16, miR-21, miR-92a) were selected and transfected into OVCAR-3 cells. With the exception of miR-16, exosomal miRNAs increased proliferation in OVCAR-3 cells (Fig. 8D) while all miRNAs protected EOC cells from PTX treatment (Fig. 8E).

## Discussion

Epithelial ovarian cancer (EOC) metastasis is a complex, multistep process involving shedding of cells from the primary tumour, intra-peritoneal dissemination, and attachment and invasion at a secondary site within the peritoneal cavity [3]. Previous work has demonstrated omentum tropism in ovarian cancer cells in a mouse model [18], while omentum-derived adipocytes and adipose stem cells can regulate gene expression in EOC cell lines in favor of survival, proliferation and chemoresistance [11, 24–26]. Omental adipocytes have been shown to provide energy for proliferating EOC via the direct transfer of fatty acids to neighbouring cancer cells [27], while suppression of this process through inhibition of CD36 or FABP4 can sensitize EOC to chemotherapy [14, 28]. Here, we further demonstrate an important role for omental adipocytes in clinically relevant aspects of EOC biology. Omentum-derived secreted factors significantly impacted EOC cell line and patient derived-spheroids proliferation (Figs. 1 and 7). Notably, this effect was apparent in omental tissue obtained from patients with both malignant and non-malignant ovarian neoplasms. The presence of pro-cancer signalling within the peritoneum highlights the importance of this microenvironment in the establishment and development of EOC metastasis. Intra-peritoneal ovarian cancer metastasis requires multiple cell types interacting in a dynamic three-dimensional environment and multicellular 3D models can, therefore, provide insights into mechanisms of disease progression [29–32]. We have developed a heterotypic spheroid model for adipocyte-EOC interactions incorporating adipocyte-derived stem cell (ADSC)-derived adipocytes and EOC cell lines (Fig. 2). Tumour spheroids incorporate aspects of a localised tumour microenvironment environment such as gradients of nutrient availability and cellular necrosis and can recapitulate phenotypic heterogeneity and mechanisms of chemoresistance which are apparent in solid tumours [33]. Furthermore, ovarian cancer metastasis has been shown to progress through the dissemination of multicellular aggregates, endogenous tumour spheroids, throughout the peritoneum [31, 34]. We found that omentum-derived factors and adipocyte co-culture had significant impact on the proliferative capacity of EOC spheroids, as well as populations grown in monolayer culture (Figs. 1 and 2).

Notably, omental tissue and adipocyte-derived paracrine signalling decreased proliferation in spheroids, in contrast to monolayer populations and SKOV-3-spheroids. To understand differences observed in response of EOC populations to adipocyte signalling, we explored the phenotypic characteristics of cell lines, specifically in relation to epithelial-to-mesenchymal transition (EMT), whereby epithelial cells undergo widespread changes in gene expression and phenotype and which has been shown to promote invasion and protect metastasizing cancer cells from therapy, while reducing proliferation [22, 35, 36]. SKOV-3 represents a post-EMT phenotype (Vimentin^+^, N-Cadherin^+^ E-Cadherin^low^) with an invasive pathology, while OVCAR-3 displayed an epithelial profile (Vimentin^−^ N-Cadherin^−^ E-Cadherin^high^) typical of a less invasive, epithelial phenotype [37–39].

Tumour spheroids have been shown to generate a heterogenous population of cells whose activity and response to therapy is determined by their position in three-dimensional space relative to spheroid architecture and by differential penetration of nutrients and other bioactive molecules, including therapeutic agents [33, 40]. OC cell lines grown in spheroid culture demonstrated significantly more heterogeneous expression of EMT markers than those grown in monolayer culture (Fig. 3). Similarly, spheroid culture has been shown to upregulate population ‘stemness’, the capacity for phenotypic change in response to signalling factors and other external stimuli [41]. We found that in response to adipocyte signalling, OVCAR-3 cells transition to a more invasive, vimentin^+^ phenotype (Fig. 4) and suggest that phenotypic heterogeneity within spheroid populations potentiates this phenotypic switch. Vimentin has previously been shown to play a central role in EMT-mediated metastasis and targeting vimentin can increase susceptibility to chemotherapy in ovarian cancer cells [39, 42, 43]. Epithelial cancer cell transition to a mesenchymal-like phenotype via EMT has been associated with poor response to chemotherapy in ovarian cancer, a major obstacle in effective treatment of advanced stage disease [44]. We explored the impact of bi-directional signalling between EOC and adipocytes on response to paclitaxel (PTX) therapy and found adipocytes acted to protect EOC cell lines from chemotherapy (Fig. 5).

Extracellular vesicles (EVs), including exosomes, have been implicated in response to chemotherapy in a wide range of cancers [45, 46], and play a significant role in signalling between malignant and non-malignant cell types [19]. Adipocytes secrete exosomes in abundance, and adipose-derived exosomes play a role in the regulation of angiogenesis, proliferation, immunity and metabolism [47]. In the tumour microenvironment, adipocyte-derived exosomes promote cancer cell proliferation and migration in breast cancer via activation of the hippo pathway [48]. Similarly, adipocyte-derived exosomes can reprogram tumour cell metabolism in melanoma through upregulation of fatty acid oxidation [49]. Here, we demonstrated that omentum-derived exosomes provide chemoprotection in EOC cells and drive EMT and invasion in OVCAR-3 via the downregulation of E-Cadherin and overexpression of N-Cadherin (Fig. 6). These data further demonstrate the capacity of adipocyte-derived exosomes to generate a pro-tumorigenic microenvironment. We validated our data using patient-derived spheroids generated from ascites samples collected from HGSOC patients, demonstrating the effect protective effect of omentum CT and omentum-exosomes against paclitaxel treatment as well as their capacity to stimulate expression of mesenchymal markers to promote an aggressive OC phenotype (Fig. 7). Interestingly, we observed that omentum-derived exosomes from patients with benign masses could also drive these changes, underscoring the crucial role of omental fat cells in facilitating cancer metastasis. Future experiments should investigate whether other fatty tissues within the peritoneal cavity, such as the mesentery, can also activate signaling pathways that promote ovarian cancer metastasis. Besides the omentum, the mesentery significantly contributes to ovarian cancer proliferation and metastasis. Mesenteric fat secretes adipokines and cytokines such as leptin, adiponectin, TNF-α, and IL-6, creating a pro-inflammatory environment that drives ovarian cancer cell proliferation and migration [50]. It also supports angiogenesis, ECM remodelling, and provides metabolic substrates to cancer cells, thereby facilitating tumour growth and dissemination [18]. Thus, both mesenteric and omental adipose tissues are pivotal in creating a microenvironment that supports cancer metastasis through complex biochemical and cellular interactions [51]. Obesity, characterized by adipose hypertrophy, is a risk factor for many cancers, including EOC [6, 7, 52] and the cargo of exosomes secreted by hypertrophic adipocytes has been shown to be drastically altered [53–55]. Obesity-induced changes in exosome content may, therefore, be a contributing factor in observed obesity and adiposity-related cancer risk. Future experiments should investigate whether other fatty tissues within the peritoneal cavity, such as the mesentery, can also activate exosome-mediated signaling pathways described here for omentum adipose tissue that promote ovarian cancer metastasis.

To better understand the pro-cancer components of adipocyte-derived exosome cargo, we performed miRNA next generation sequencing on exosomes secreted by primary omental tissue. Exosome-derived microRNAs have an impact on cancer development, progression, mortality, and resistance to therapy [23, 45, 56, 57]. We identified highly abundant miRNAs in patients with both benign and malignant neoplasms (Fig. 7). Pathway analysis revealed signalling networks related to ovarian cancer as well as breast, prostate, bladder, and endometrial cancers. Adipocytes have been shown to promote cancer development in breast [16, 58, 59] and prostate cancer [60, 61], while obesity is a risk factor for endometrial cancer [12, 62, 63]. The exosome miRNA-regulated signalling networks identified here may therefore play a wider-reaching role in the development of the metastatic adipose niche and cancer development, especially those cancers where increased adiposity is a known risk factor. Furthermore, prolactin (PRL) signalling, identified here through pathway analysis, is upregulated in numerous hormone-dependent cancers including ovarian, breast, prostate and endometrial cancer and is linked to cancer via activation of JAK/STAT, AKT and MAPK pathways [64–69]. In EOC, PRL-JAK-STAT pathway signalling can lead to phosphorylation of STAT3 and STAT5 [66], connecting PRL signalling to EMT, EOC cancer stemness and resistance to therapy [34, 70–72].

To validate the role of adipocyte-derived exosome miRNAs in EOC we assessed the impact of miR-21, let-7b, miR-16 and miR-92a on OVCAR-3 proliferation and response to therapy and found a chemoprotective effect for each miRNA. Previous work has demonstrated the transfer of miR-21 from adipocytes to EOC cell lines and shown that miR-21 can lower sensitivity to PTX in cancer cells in in vitro and in vivo models [21]. This previous study suggested miR-21 exerts its chemoprotective effects via downregulation of APAF1, while miR-21 can also promote cisplatin resistance in EOC by negatively targeting the tumour suppressor PTEN [73]. Exosomal miR-21, derived from cancer associated fibroblasts, was shown also to activate STAT3 and promote cisplatin resistance in oesophageal squamous cell carcinoma [74]. miR-92a is part of the oncogenic miR-72-92 cluster and is highly expressed in a number of cancers, including in EOC patient serum [75, 76], and its targets include E-Cadherin [77]. The role of Let-7b in reducing PTX cytotoxicity is less clear as the Let-7 family of miRNAs have previously been shown to act as tumour suppressors in ovarian cancer [78–80] and let-7b is relatively less abundant in exosomes derived from patients with ovarian malignancies (Fig. 8A) as seen elsewhere [80, 81]. However, transfection of OVCAR-3 with let-7b miRNA increased proliferation and protection from PTX therapy (Fig. 8D, E). Alongside the data presented here, a meta-analysis of transcriptomes of 1,170 patients with high grade ovarian cancer showed let-7b was associated with poor survival rates, implicating let-7b in resistance to therapy [82]. Previous work has elucidated the role in the tumour-derived exosomes in the formation of the pre-metastatic niche [83] while here we demonstrate the capacity for adipocyte-derived exosomes to generate a pro-tumorigenic, chemoprotective environment via miRNA signalling. More importantly, here we show that -derived adipocytes from non-cancer patients also contain key pro-oncogenic mediators of cancer proliferation, invasion and response to treatment, highlighting the importance of increased adiposity as known cancer risk factor. Therapies which aim to target cells within adipose tissue in order to suppress cancer have shown promise in animal models may provide a means to modulate and inhibit cancer-specific signalling and metabolism [84, 85]. Currently, obese and non-obese patients receive the same cancer treatment, despite apparent differences in risk and outcome [8]. A more comprehensive understanding of the mechanisms through which the adipose microenvironment drives pathogenesis and chemoresistance will enable tailored therapies which target specific pro-cancer signalling in healthy and obese or dysregulated adipose tissue.

## Conclusions

Taken together, these data suggest an important role for adipose-derived exosomes in EOC metastasis. Transformed epithelial cells in the primary ovarian or fallopian tube microenvironment must undergo drastic phenotypic changes to permit dissemination into the peritoneum and invasion of peritoneal organs such as the omentum. We propose an exosome-derived miRNA signalling mechanism, acting at distance across the peritoneum, by which adipose tissue may act to promote ovarian cancer cell motility and invasion via EMT while protecting cancer cells from therapy. Experimental therapeutics targeting cellular and molecular mechanisms through which adipocytes drive cancer cell proliferation and progression could reduce the increased cancer risk associated with obesity and could improve the efficacy of current anticancer treatments.

## Electronic supplementary material

Below is the link to the electronic supplementary material.

Supplementary Material 1

Supplementary Material 2

## Data Availability

Anonymized data sets used in this study are available from the authors upon request.

## Abbreviations

ADSC: Adipose-derived stem cell
EMT: Epithelial-to-mesenchymal transition
ELISA: Enzyme-linked immunosorbent assay
EOC: Epithelial ovarian cancer
EVs: Extracellular vesicles
HGSOC: High-grade serous ovarian carcinoma
IL: 6-Interleukin-6
IL: 8-Interleukin-8
miRNA: MicroRNA
OT: CM-Omental tissue-conditioned media
PRL: Prolactin
PTX: Paclitaxel
UCM: Unconditioned media

## References

[1] Matulonis UA, Sood AK, Fallowfield L, Howitt BE, Sehouli J, Karlan BY. Ovarian cancer. Nat Reviews Disease Primers. 2016;2(1):1–22. 10.1038/nrdp.2016.61 PMC7290868 27558151

[2] Shaik B, Zafar T, Balasubramanian K, Gupta SP. An overview of ovarian cancer: molecular processes involved and development of target-based chemotherapeutics. Curr Top Med Chem. 2021;21(4):329–46. 33183204 10.2174/1568026620999201111155426

[3] Lengyel E. Ovarian cancer development and metastasis. Am J Pathol. 2010;177(3):1053–64. 20651229 10.2353/ajpath.2010.100105 PMC2928939

[4] Meza-Perez S, Randall TD. Immunological functions of the omentum. Trends Immunol. 2017;38(7):526–36. 28579319 10.1016/j.it.2017.03.002 PMC5812451

[5] Yeung T-L, Leung CS, Yip K-P, Yeung CLA, Wong ST, Mok SC. Cellular and molecular processes in ovarian cancer metastasis. A review in the theme: cell and molecular processes in cancer metastasis. Am J Physiology-Cell Physiol. 2015. 10.1152/ajpcell.00188.2015 PMC4593771 26224579

[6] Nagle C, Dixon S, Jensen A, Kjaer S, Modugno F, DeFazio A, et al. Obesity and survival among women with ovarian cancer: results from the ovarian cancer association consortium. Br J Cancer. 2015;113(5):817–26. 26151456 10.1038/bjc.2015.245 PMC4559823

[7] Olsen CM, Green AC, Whiteman DC, Sadeghi S, Kolahdooz F, Webb PM. Obesity and the risk of epithelial ovarian cancer: a systematic review and meta-analysis. Eur J Cancer. 2007;43(4):690–709. 17223544 10.1016/j.ejca.2006.11.010

[8] Lengyel E, Makowski L, DiGiovanni J, Kolonin MG. Cancer as a matter of fat: the crosstalk between adipose tissue and tumors. Trends cancer. 2018;4(5):374–84. 29709261 10.1016/j.trecan.2018.03.004 PMC5932630

[9] Gunderson CC, Ding K, Dvorak J, Moore KN, McMeekin DS, Benbrook DM. The pro-inflammatory effect of obesity on high grade serous ovarian cancer. Gynecol Oncol. 2016;143(1):40–5. 27423378 10.1016/j.ygyno.2016.07.103

[10] Giornelli GH. Management of relapsed ovarian cancer: a review. Springerplus. 2016;5(1):1197. 27516935 10.1186/s40064-016-2660-0 PMC4963348

[11] Nowicka A, Marini FC, Solley TN, Elizondo PB, Zhang Y, Sharp HJ, et al. Human omental-derived adipose stem cells increase ovarian cancer proliferation, migration, and chemoresistance. PLoS ONE. 2013;8(12):e81859. 24312594 10.1371/journal.pone.0081859 PMC3847080

[12] Salimian Rizi B, Caneba C, Nowicka A, Nabiyar AW, Liu X, Chen K, et al. Nitric oxide mediates metabolic coupling of omentum-derived adipose stroma to ovarian and endometrial cancer cells. Cancer Res. 2015;75(2):456–71. 25425006 10.1158/0008-5472.CAN-14-1337

[13] Yang J, Zaman MM, Vlasakov I, Roy R, Huang L, Martin CR, et al. Adipocytes promote ovarian cancer chemoresistance. Sci Rep. 2019;9(1):13316. 31527632 10.1038/s41598-019-49649-1 PMC6746782

[14] Mukherjee A, Chiang C-Y, Daifotis HA, Nieman KM, Fahrmann JF, Lastra RR, et al. Adipocyte-induced FABP4 expression in ovarian cancer cells promotes metastasis and mediates carboplatin resistance. Cancer Res. 2020;80(8):1748–61. 32054768 10.1158/0008-5472.CAN-19-1999 PMC10656748

[15] Nath S, Pigula M, Khan AP, Hanna W, Ruhi MK, Dehkordy FM, et al. Flow-induced shear stress confers resistance to carboplatin in an adherent three-dimensional model for ovarian cancer: a role for EGFR-targeted photoimmunotherapy informed by physical stress. J Clin Med. 2020;9(4):924. 32231055 10.3390/jcm9040924 PMC7230263

[16] Wang YY, Attané C, Milhas D, Dirat B, Dauvillier S, Guerard A et al. Mammary adipocytes stimulate breast cancer invasion through metabolic remodeling of tumor cells. JCI Insight. 2017;2(4). 10.1172/jci.insight.87489 PMC5313068 28239646

[17] Li J, Condello S, Thomes-Pepin J, Ma X, Xia Y, Hurley TD, et al. Lipid desaturation is a metabolic marker and therapeutic target of ovarian cancer stem cells. Cell Stem Cell. 2017;20(3):303–14. e5. 28041894 10.1016/j.stem.2016.11.004 PMC5337165

[18] Nieman KM, Kenny HA, Penicka CV, Ladanyi A, Buell-Gutbrod R, Zillhardt MR, et al. Adipocytes promote ovarian cancer metastasis and provide energy for rapid tumor growth. Nat Med. 2011;17(11):1498–503. 22037646 10.1038/nm.2492 PMC4157349

[19] Wortzel I, Dror S, Kenific CM, Lyden D. Exosome-mediated metastasis: communication from a distance. Dev Cell. 2019;49(3):347–60. 31063754 10.1016/j.devcel.2019.04.011

[20] Yokoi A, Yoshioka Y, Yamamoto Y, Ishikawa M, Ikeda S-i, Kato T, et al. Malignant extracellular vesicles carrying MMP1 mRNA facilitate peritoneal dissemination in ovarian cancer. Nat Commun. 2017;8(1):14470. 28262727 10.1038/ncomms14470 PMC5343481

[21] Au Yeung CL, Co N-N, Tsuruga T, Yeung T-L, Kwan S-Y, Leung CS, et al. Exosomal transfer of stroma-derived miR21 confers paclitaxel resistance in ovarian cancer cells through targeting APAF1. Nat Commun. 2016;7(1):11150. 27021436 10.1038/ncomms11150 PMC4820618

[22] Ahmed N, Abubaker K, Findlay J, Quinn M. Epithelial mesenchymal transition and cancer stem cell-like phenotypes facilitate chemoresistance in recurrent ovarian cancer. Curr Cancer Drug Targets. 2010;10(3):268–78. 20370691 10.2174/156800910791190175

[23] Saburi A, Kahrizi MS, Naghsh N, Etemadi H, İlhan A, Adili A, et al. A comprehensive survey into the role of microRNAs in ovarian cancer chemoresistance; an updated overview. J Ovarian Res. 2022;15(1):1–14. 35799305 10.1186/s13048-022-01012-1 PMC9264529

[24] Miranda F, Mannion D, Liu S, Zheng Y, Mangala LS, Redondo C, et al. Salt-inducible kinase 2 couples ovarian cancer cell metabolism with survival at the adipocyte-rich metastatic niche. Cancer Cell. 2016;30(2):273–89. 27478041 10.1016/j.ccell.2016.06.020

[25] Ladanyi A, Mukherjee A, Kenny HA, Johnson A, Mitra AK, Sundaresan S, et al. Adipocyte-induced CD36 expression drives ovarian cancer progression and metastasis. Oncogene. 2018;37(17):2285–301. 29398710 10.1038/s41388-017-0093-z PMC5920730

[26] Zhang Y, Dong W, Wang J, Cai J, Wang Z. Human omental adipose-derived mesenchymal stem cell-conditioned medium alters the proteomic profile of epithelial ovarian cancer cell lines in vitro. OncoTargets Therapy. 2017;10:1655. 28360526 10.2147/OTT.S129502 PMC5364023

[27] Nieman KM, Romero IL, Van Houten B, Lengyel E. Adipose tissue and adipocytes support tumorigenesis and metastasis. Biochim et Biophys Acta (BBA)-Molecular Cell Biology Lipids. 2013;1831(10):1533–41. 10.1016/j.bbalip.2013.02.010 PMC3742583 23500888

[28] Chen RR, Yung MM, Xuan Y, Zhan S, Leung LL, Liang RR, et al. Targeting of lipid metabolism with a metabolic inhibitor cocktail eradicates peritoneal metastases in ovarian cancer cells. Commun Biology. 2019;2(1):281. 10.1038/s42003-019-0508-1 PMC6668395 31372520

[29] Matte I, Legault CM, Garde-Granger P, Laplante C, Bessette P, Rancourt C, et al. Mesothelial cells interact with tumor cells for the formation of ovarian cancer multicellular spheroids in peritoneal effusions. Clin Exp Metastasis. 2016;33:839–52. 27612856 10.1007/s10585-016-9821-y

[30] Shishido A, Mori S, Yokoyama Y, Hamada Y, Minami K, Qian Y, et al. Mesothelial cells facilitate cancer stem–like properties in spheroids of ovarian cancer cells. Oncol Rep. 2018;40(4):2105–14. 30066911 10.3892/or.2018.6605

[31] Gao Q, Yang Z, Xu S, Li X, Yang X, Jin P, et al. Heterotypic CAF-tumor spheroids promote early peritoneal metastasis of ovarian cancer. J Exp Med. 2019;216(3):688–703. 30710055 10.1084/jem.20180765 PMC6400537

[32] Yin M, Li X, Tan S, Zhou HJ, Ji W, Bellone S, et al. Tumor-associated macrophages drive spheroid formation during early transcoelomic metastasis of ovarian cancer. J Clin Investig. 2016;126(11):4157–73. 27721235 10.1172/JCI87252 PMC5096908

[33] Costa EC, Moreira AF, de Melo-Diogo D, Gaspar VM, Carvalho MP, Correia IJ. 3D tumor spheroids: an overview on the tools and techniques used for their analysis. Biotechnol Adv. 2016;34(8):1427–41. 27845258 10.1016/j.biotechadv.2016.11.002

[34] Latifi A, Luwor RB, Bilandzic M, Nazaretian S, Stenvers K, Pyman J et al. Isolation and characterization of tumor cells from the ascites of ovarian cancer patients: molecular phenotype of chemoresistant ovarian tumors; 2012. 10.1371/journal.pone.0046858 PMC3466197 23056490

[35] Iwatsuki M, Mimori K, Yokobori T, Ishi H, Beppu T, Nakamori S, et al. Epithelial–mesenchymal transition in cancer development and its clinical significance. Cancer Sci. 2010;101(2):293–9. 19961486 10.1111/j.1349-7006.2009.01419.x PMC11159985

[36] Haslehurst AM, Koti M, Dharsee M, Nuin P, Evans K, Geraci J, et al. EMT transcription factors snail and slug directly contribute to cisplatin resistance in ovarian cancer. BMC Cancer. 2012;12:1–10. 22429801 10.1186/1471-2407-12-91 PMC3342883

[37] Sawada K, Mitra AK, Radjabi AR, Bhaskar V, Kistner EO, Tretiakova M, et al. Loss of E-cadherin promotes ovarian cancer metastasis via α5-integrin, which is a therapeutic target. Cancer Res. 2008;68(7):2329–39. 18381440 10.1158/0008-5472.CAN-07-5167 PMC2665934

[38] Moreno-Bueno G, Peinado H, Molina P, Olmeda D, Cubillo E, Santos V, et al. The morphological and molecular features of the epithelial-to-mesenchymal transition. Nat Protoc. 2009;4(11):1591–613. 19834475 10.1038/nprot.2009.152

[39] Li X, Yang J, Wang X, Liang J, Xing H. Role of TWIST2, E-cadherin and Vimentin in epithelial ovarian carcinogenesis and prognosis and their interaction in cancer progression. Eur J Gynaecol Oncol. 2016;37(1):100–8. 27048119

[40] Shield K, Ackland ML, Ahmed N, Rice GE. Multicellular spheroids in ovarian cancer metastases: Biology and pathology. Gynecol Oncol. 2009;113(1):143–8. 19135710 10.1016/j.ygyno.2008.11.032

[41] Liao J, Qian F, Tchabo N, Mhawech-Fauceglia P, Beck A, Qian Z, et al. Ovarian cancer spheroid cells with stem cell-like properties contribute to tumor generation, metastasis and chemotherapy resistance through hypoxia-resistant metabolism. PLoS ONE. 2014;9(1):e84941. 24409314 10.1371/journal.pone.0084941 PMC3883678

[42] Usman S, Waseem NH, Nguyen TKN, Mohsin S, Jamal A, Teh M-T, et al. Vimentin is at the heart of epithelial mesenchymal transition (EMT) mediated metastasis. Cancers. 2021;13(19):4985. 34638469 10.3390/cancers13194985 PMC8507690

[43] Han X, Zhou Y, You Y, Lu J, Wang L, Hou H, et al. TET1 promotes cisplatin-resistance via demethylating the vimentin promoter in ovarian cancer. Cell Biol Int. 2017;41(4):405–14. 28150354 10.1002/cbin.10734

[44] Pujade-Lauraine E, Banerjee S, Pignata S. Management of platinum-resistant, relapsed epithelial ovarian cancer and new drug perspectives. J Clin Oncol. 2019;37(27):2437–48. 31403868 10.1200/JCO.19.00194

[45] Bach DH, Hong JY, Park HJ, Lee SK. The role of exosomes and miRNAs in drug-resistance of cancer cells. Int J Cancer. 2017;141(2):220–30. 28240776 10.1002/ijc.30669

[46] Tian W, Lei N, Zhou J, Chen M, Guo R, Qin B, et al. Extracellular vesicles in ovarian cancer chemoresistance, metastasis, and immune evasion. Cell Death Dis. 2022;13(1):64. 35042862 10.1038/s41419-022-04510-8 PMC8766448

[47] Jafari N, Kolla M, Meshulam T, Shafran JS, Qiu Y, Casey AN, et al. Adipocyte-derived exosomes may promote breast cancer progression in type 2 diabetes. Sci Signal. 2021;14(710):eabj2807. 34813359 10.1126/scisignal.abj2807 PMC8765301

[48] Wang S, Su X, Xu M, Xiao X, Li X, Li H, et al. Exosomes secreted by mesenchymal stromal/stem cell-derived adipocytes promote breast cancer cell growth via activation of Hippo signaling pathway. Stem Cell Res Ther. 2019;10:1–12. 30971292 10.1186/s13287-019-1220-2 PMC6458638

[49] Lazar I, Clement E, Dauvillier S, Milhas D, Ducoux-Petit M, LeGonidec S, et al. Adipocyte exosomes promote Melanoma aggressiveness through fatty acid oxidation: a novel mechanism linking obesity and CancerAdipocyte exosomes: a new link between obesity and Cancer. Cancer Res. 2016;76(14):4051–7. 27216185 10.1158/0008-5472.CAN-16-0651

[50] Park J, Morley TS, Kim M, Clegg DJ, Scherer PE. Obesity and cancer—mechanisms underlying tumour progression and recurrence. Nat Reviews Endocrinol. 2014;10(8):455–65. 10.1038/nrendo.2014.94 PMC4374431 24935119

[51] Brown KA, Scherer PE. Update on adipose tissue and cancer. Endocr Rev. 2023;44(6):961–74. 37260403 10.1210/endrev/bnad015 PMC10638602

[52] Wolin KY, Carson K, Colditz GA. Obesity and cancer. Oncologist. 2010;15(6):556–65. 20507889 10.1634/theoncologist.2009-0285 PMC3227989

[53] Zhang B, Yang Y, Xiang L, Zhao Z, Ye R. Adipose-derived exosomes: a novel adipokine in obesity‐associated diabetes. J Cell Physiol. 2019;234(10):16692–702. 30807657 10.1002/jcp.28354

[54] Sano S, Izumi Y, Yamaguchi T, Yamazaki T, Tanaka M, Shiota M, et al. Lipid synthesis is promoted by hypoxic adipocyte-derived exosomes in 3T3-L1 cells. Biochem Biophys Res Commun. 2014;445(2):327–33. 24513287 10.1016/j.bbrc.2014.01.183

[55] Wen Z, Li J, Fu Y, Zheng Y, Ma M, Wang C. Hypertrophic adipocyte–derived exosomal mir-802‐5p contributes to Insulin Resistance in Cardiac myocytes through Targeting HSP60. Obesity. 2020;28(10):1932–40. 32844579 10.1002/oby.22932

[56] Yang H, Kong W, He L, Zhao J-J, O’Donnell JD, Wang J, et al. MicroRNA expression profiling in human ovarian cancer: miR-214 induces cell survival and cisplatin resistance by targeting PTEN. Cancer Res. 2008;68(2):425–33. 18199536 10.1158/0008-5472.CAN-07-2488

[57] Koutsaki M, Spandidos DA, Zaravinos A. Epithelial–mesenchymal transition-associated miRNAs in ovarian carcinoma, with highlight on the miR-200 family: prognostic value and prospective role in ovarian cancer therapeutics. Cancer Lett. 2014;351(2):173–81. 24952258 10.1016/j.canlet.2014.05.022

[58] Dirat B, Bochet L, Dabek M, Daviaud D, Dauvillier S, Majed B, et al. Cancer-associated adipocytes exhibit an activated phenotype and contribute to breast cancer invasion. Cancer Res. 2011;71(7):2455–65. 21459803 10.1158/0008-5472.CAN-10-3323

[59] Wu Q, Li B, Li Z, Li J, Sun S, Sun S. Cancer-associated adipocytes: key players in breast cancer progression. J Hematol Oncol. 2019;12:1–15. 31500658 10.1186/s13045-019-0778-6 PMC6734503

[60] Laurent V, Guérard A, Mazerolles C, Le Gonidec S, Toulet A, Nieto L, et al. Periprostatic adipocytes act as a driving force for prostate cancer progression in obesity. Nat Commun. 2016;7(1):10230. 26756352 10.1038/ncomms10230 PMC4729927

[61] Fujita K, Hayashi T, Matsushita M, Uemura M, Nonomura N. Obesity, inflammation, and prostate cancer. J Clin Med. 2019;8(2):201. 30736371 10.3390/jcm8020201 PMC6406330

[62] Nakamura K, Hongo A, Kodama J, Hiramatsu Y. Fat accumulation in adipose tissues as a risk factor for the development of endometrial cancer. Oncol Rep. 2011;26(1):65–71. 21491090 10.3892/or.2011.1259

[63] Moukarzel LA, Ferrando L, Stylianou A, Lobaugh S, Wu M, Nobre SP, et al. Impact of obesity and white adipose tissue inflammation on the omental microenvironment in endometrial cancer. Cancer. 2022;128(18):3297–309. 35793549 10.1002/cncr.34356 PMC9976596

[64] Ben-Jonathan N, Liby K, McFarland M, Zinger M. Prolactin as an autocrine/paracrine growth factor in human cancer. Trends Endocrinol Metabolism. 2002;13(6):245–50. 10.1016/S1043-2760(02)00603-3 12128285

[65] Vonderhaar B. Prolactin involvement in breast cancer. Endocrine-related Cancer. 1999;6(3):389–404. 10516853 10.1677/erc.0.0060389

[66] Alkharusi A, AlMuslahi A, AlBalushi N, AlAjmi R, AlRawahi S, AlFarqani A, et al. Connections between prolactin and ovarian cancer. PLoS ONE. 2021;16(8):e0255701. 34358244 10.1371/journal.pone.0255701 PMC8345882

[67] Yurkovetsky Z, Ta’asan S, Skates S, Rand A, Lomakin A, Linkov F, et al. Development of multimarker panel for early detection of endometrial cancer. High diagnostic power of prolactin. Gynecol Oncol. 2007;107(1):58–65. 17659325 10.1016/j.ygyno.2007.05.041 PMC2777971

[68] Levina VV, Nolen B, Su Y, Godwin AK, Fishman D, Liu J, et al. Biological significance of prolactin in gynecologic cancers. Cancer Res. 2009;69(12):5226–33. 19491263 10.1158/0008-5472.CAN-08-4652 PMC2918393

[69] Jacobson EM, Hugo ER, Borcherding DC, Ben-Jonathan N. Prolactin in breast and prostate cancer: molecular and genetic perspectives. Discov Med. 2011;11(59):315–24. 21524385

[70] Yue P, Zhang X, Paladino D, Sengupta B, Ahmad S, Holloway RW, et al. Hyperactive EGF receptor, Jaks and Stat3 signaling promote enhanced colony-forming ability, motility and migration of cisplatin-resistant ovarian cancer cells. Oncogene. 2012;31(18):2309–22. 21909139 10.1038/onc.2011.409 PMC3245777

[71] Colomiere M, Ward AC, Riley C, Trenerry MK, Cameron-Smith D, Findlay J, et al. Cross talk of signals between EGFR and IL-6R through JAK2/STAT3 mediate epithelial–mesenchymal transition in ovarian carcinomas. Br J Cancer. 2009;100(1):134–44. 19088723 10.1038/sj.bjc.6604794 PMC2634691

[72] Abubaker K, Luwor RB, Escalona R, McNally O, Quinn MA, Thompson EW, et al. Targeted disruption of the JAK2/STAT3 pathway in combination with systemic administration of paclitaxel inhibits the priming of ovarian cancer stem cells leading to a reduced tumor burden. Front Oncol. 2014;4:75. 24782986 10.3389/fonc.2014.00075 PMC3988380

[73] Yu X, Chen Y, Tian R, Li J, Li H, Lv T, et al. miRNA-21 enhances chemoresistance to cisplatin in epithelial ovarian cancer by negatively regulating PTEN. Oncol Lett. 2017;14(2):1807–10. 28789414 10.3892/ol.2017.6324 PMC5529949

[74] Zhao Q, Huang L, Qin G, Qiao Y, Ren F, Shen C, et al. Cancer-associated fibroblasts induce monocytic myeloid-derived suppressor cell generation via IL-6/exosomal miR-21-activated STAT3 signaling to promote cisplatin resistance in esophageal squamous cell carcinoma. Cancer Lett. 2021;518:35–48. 34139285 10.1016/j.canlet.2021.06.009

[75] Guo F, Tian J, Lin Y, Jin Y, Wang L, Cui M. Serum microRNA-92 expression in patients with ovarian epithelial carcinoma. J Int Med Res. 2013;41(5):1456–61. 23963852 10.1177/0300060513487652

[76] Olive V, Jiang I, He L. mir-17-92, a cluster of miRNAs in the midst of the cancer network. Int J Biochem Cell Biol. 2010;42(8):1348–54. 20227518 10.1016/j.biocel.2010.03.004 PMC3681296

[77] Chen Z-l, Zhao X-h, Wang J-w, Li B-z, Wang Z, Sun J, et al. microRNA-92a promotes lymph node metastasis of human esophageal squamous cell carcinoma via E-cadherin. J Biol Chem. 2011;286(12):10725–34. 21148309 10.1074/jbc.M110.165654 PMC3060523

[78] Busch B, Bley N, Müller S, Glaß M, Misiak D, Lederer M, et al. The oncogenic triangle of HMGA2, LIN28B and IGF2BP1 antagonizes tumor-suppressive actions of the let-7 family. Nucleic Acids Res. 2016;44(8):3845–64. 26917013 10.1093/nar/gkw099 PMC4856984

[79] Nam EJ, Yoon H, Kim SW, Kim H, Kim YT, Kim JH, et al. MicroRNA expression profiles in serous ovarian carcinoma. Clin Cancer Res. 2008;14(9):2690–5. 18451233 10.1158/1078-0432.CCR-07-1731

[80] Yu Z, Kim J, He L, Creighton CJ, Gunaratne PH, Hawkins SM, et al. Functional analysis of miR-34c as a putative tumor suppressor in high-grade serous ovarian cancer. Biol Reprod. 2014;91(5):113. 25273528 10.1095/biolreprod.114.121988 PMC6366460

[81] Yamamoto CM, Oakes ML, Murakami T, Muto MG, Berkowitz RS, Ng S-W. Comparison of benign peritoneal fluid-and ovarian cancer ascites-derived extracellular vesicle RNA biomarkers. J Ovarian Res. 2018;11:1–9. 29499737 10.1186/s13048-018-0391-2 PMC5834862

[82] Tang Z, Ow GS, Thiery JP, Ivshina AV, Kuznetsov VA. Meta-analysis of transcriptome reveals let‐7b as an unfavorable prognostic biomarker and predicts molecular and clinical subclasses in high‐grade serous ovarian carcinoma. Int J Cancer. 2014;134(2):306–18. 23825028 10.1002/ijc.28371

[83] Feng W, Dean DC, Hornicek FJ, Shi H, Duan Z. Exosomes promote pre-metastatic niche formation in ovarian cancer. Mol Cancer. 2019;18:1–11. 31409361 10.1186/s12943-019-1049-4 PMC6691526

[84] Daquinag AC, Dadbin A, Snyder B, Wang X, Sahin AA, Ueno NT, et al. Non-glycanated decorin is a drug target on human adipose stromal cells. Mol Therapy-Oncolytics. 2017;6:1–9. 10.1016/j.omto.2017.05.003 PMC5458115 28607949

[85] Daquinag AC, Tseng C, Zhang Y, Amaya-Manzanares F, Florez F, Dadbin A, et al. Targeted proapoptotic peptides depleting adipose stromal cells inhibit tumor growth. Mol Ther. 2016;24(1):34–40. 26316391 10.1038/mt.2015.155 PMC4754543

