# Supplementary material for: Adipocyte derived exosomes promote cell invasion and challenge paclitaxel efficacy in ovarian cancer
Source: Cell Commun Signal. 2024 Sep 16;22:443. doi: 10.1186/s12964-024-01806-4 (PMC11404028; doi:10.1186/s12964-024-01806-4)
Supplement: Supplementary file 1 — Supplementary Material 1 [file 12964_2024_1806_MOESM1_ESM.pptx]

## Slide 1
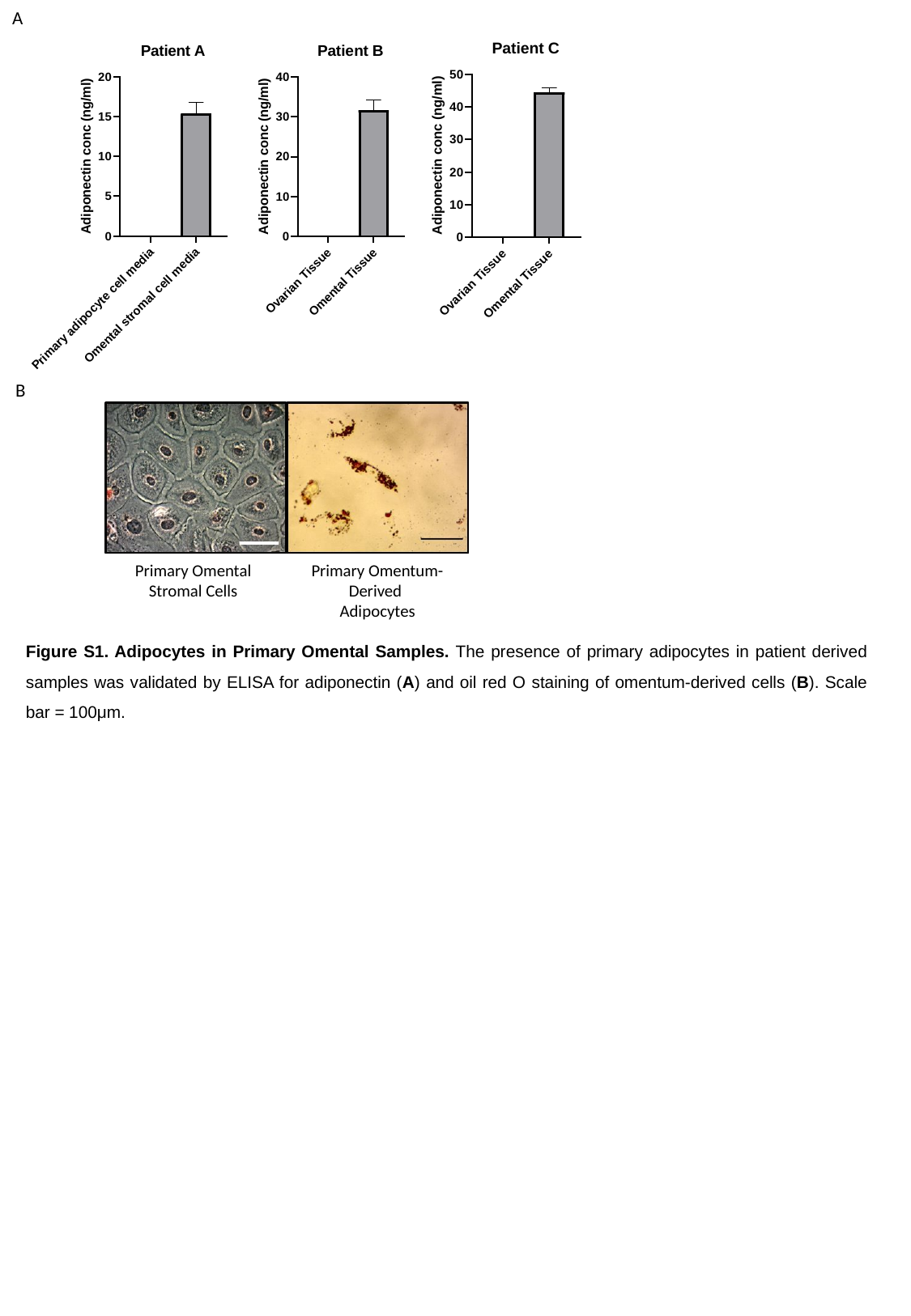

A
B
Primary Omental Stromal Cells
Primary Omentum-Derived
Adipocytes
Figure S1. Adipocytes in Primary Omental Samples. The presence of primary adipocytes in patient derived samples was validated by ELISA for adiponectin (A) and oil red O staining of omentum-derived cells (B). Scale bar = 100μm.

## Slide 2
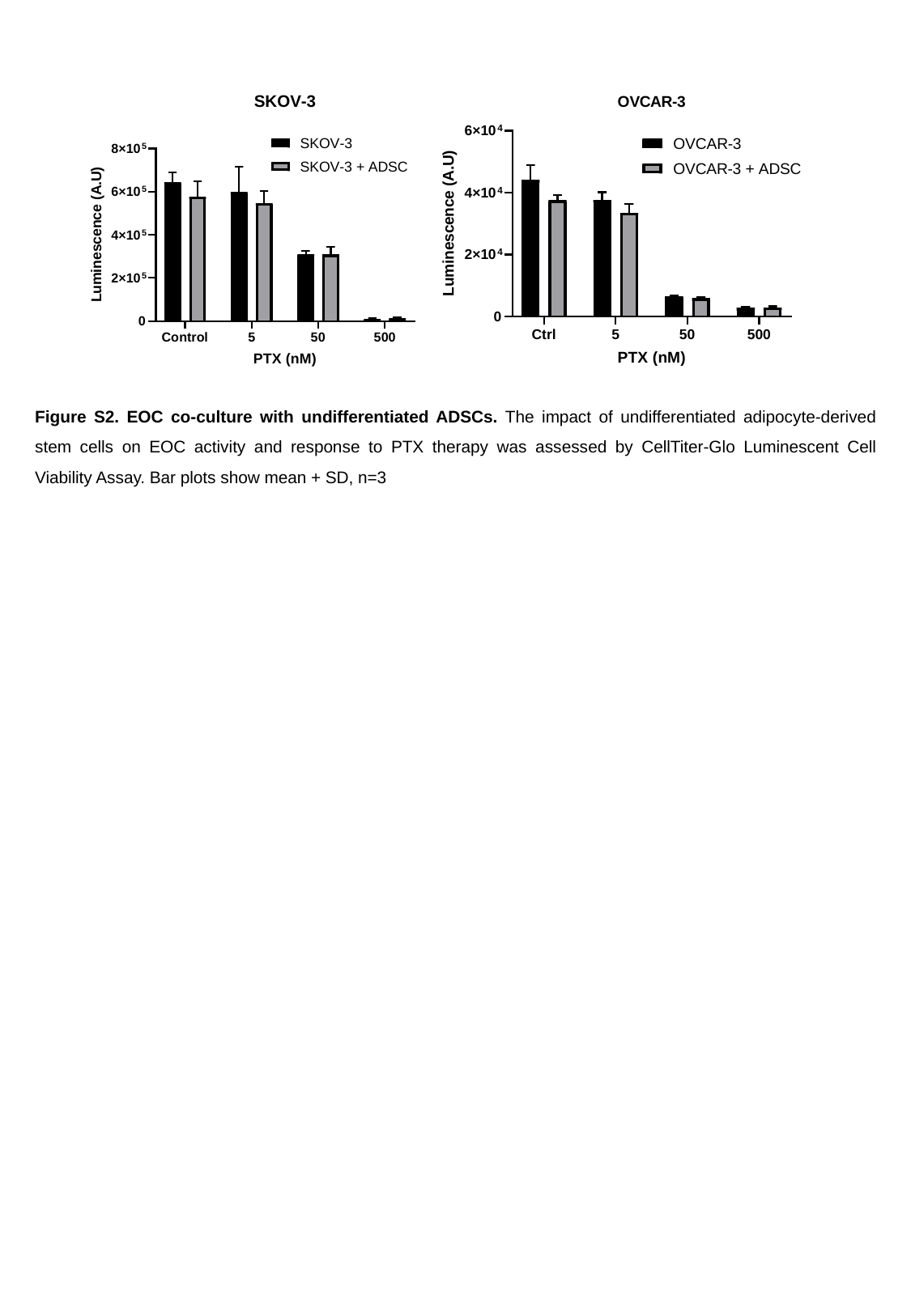

Figure S2. EOC co-culture with undifferentiated ADSCs. The impact of undifferentiated adipocyte-derived stem cells on EOC activity and response to PTX therapy was assessed by CellTiter-Glo Luminescent Cell Viability Assay. Bar plots show mean + SD, n=3

## Slide 3
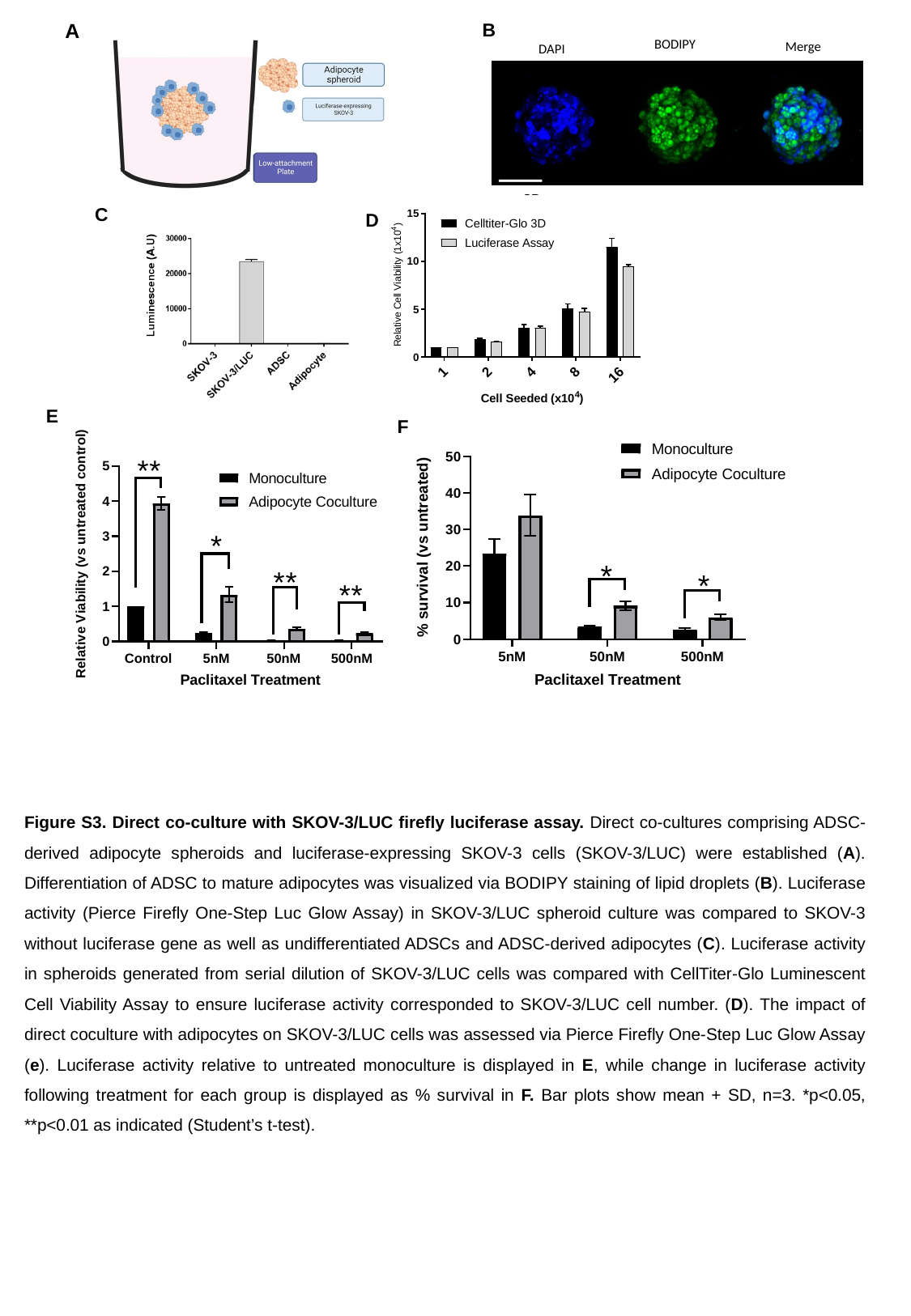

A
B
BODIPY
Merge
DAPI
C
D
E
F
Figure S3. Direct co-culture with SKOV-3/LUC firefly luciferase assay. Direct co-cultures comprising ADSC-derived adipocyte spheroids and luciferase-expressing SKOV-3 cells (SKOV-3/LUC) were established (A). Differentiation of ADSC to mature adipocytes was visualized via BODIPY staining of lipid droplets (B). Luciferase activity (Pierce Firefly One-Step Luc Glow Assay) in SKOV-3/LUC spheroid culture was compared to SKOV-3 without luciferase gene as well as undifferentiated ADSCs and ADSC-derived adipocytes (C). Luciferase activity in spheroids generated from serial dilution of SKOV-3/LUC cells was compared with CellTiter-Glo Luminescent Cell Viability Assay to ensure luciferase activity corresponded to SKOV-3/LUC cell number. (D). The impact of direct coculture with adipocytes on SKOV-3/LUC cells was assessed via Pierce Firefly One-Step Luc Glow Assay (e). Luciferase activity relative to untreated monoculture is displayed in E, while change in luciferase activity following treatment for each group is displayed as % survival in F. Bar plots show mean + SD, n=3. *p<0.05, **p<0.01 as indicated (Student’s t-test).

## Slide 4
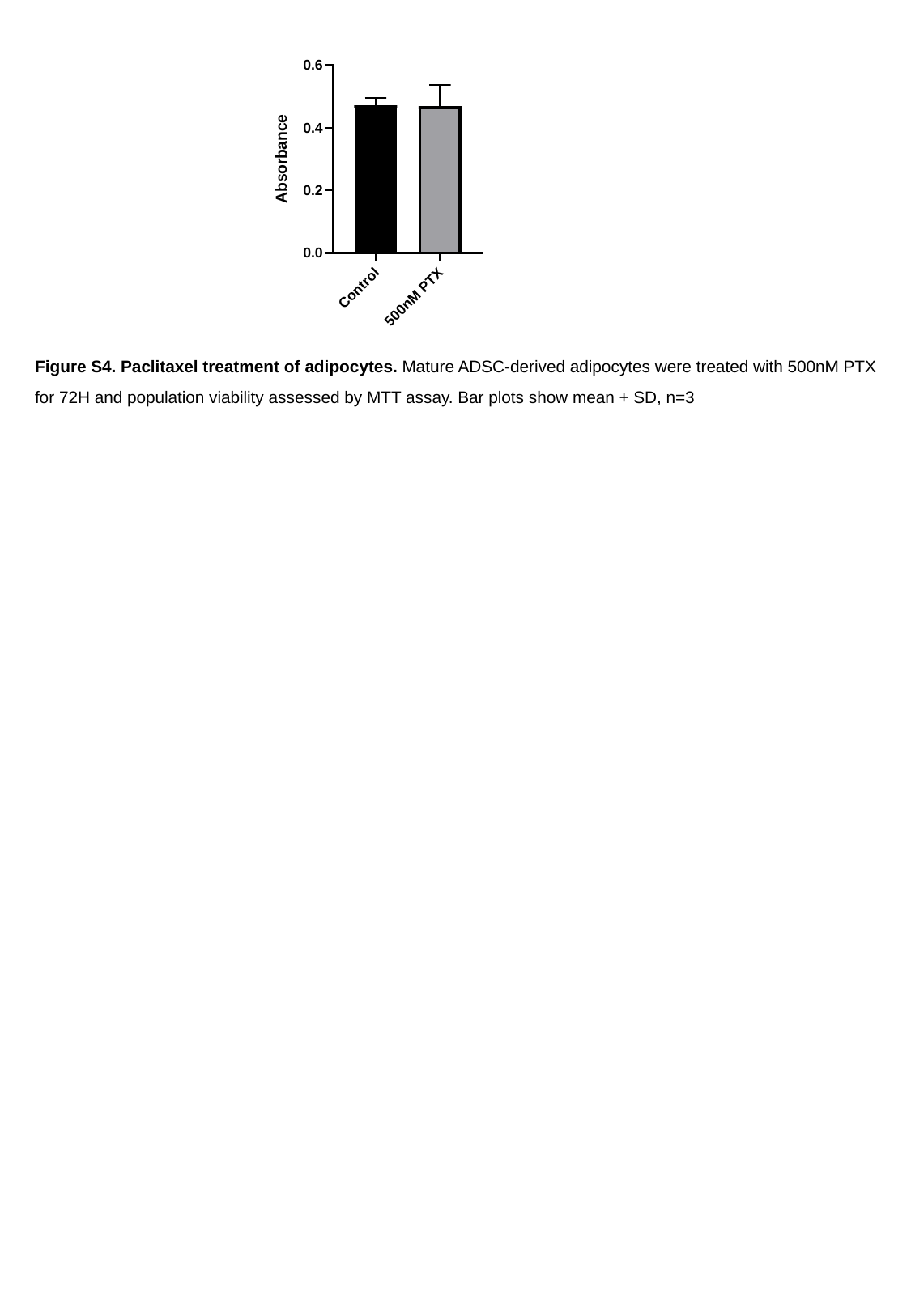

Figure S4. Paclitaxel treatment of adipocytes. Mature ADSC-derived adipocytes were treated with 500nM PTX for 72H and population viability assessed by MTT assay. Bar plots show mean + SD, n=3

## Slide 5
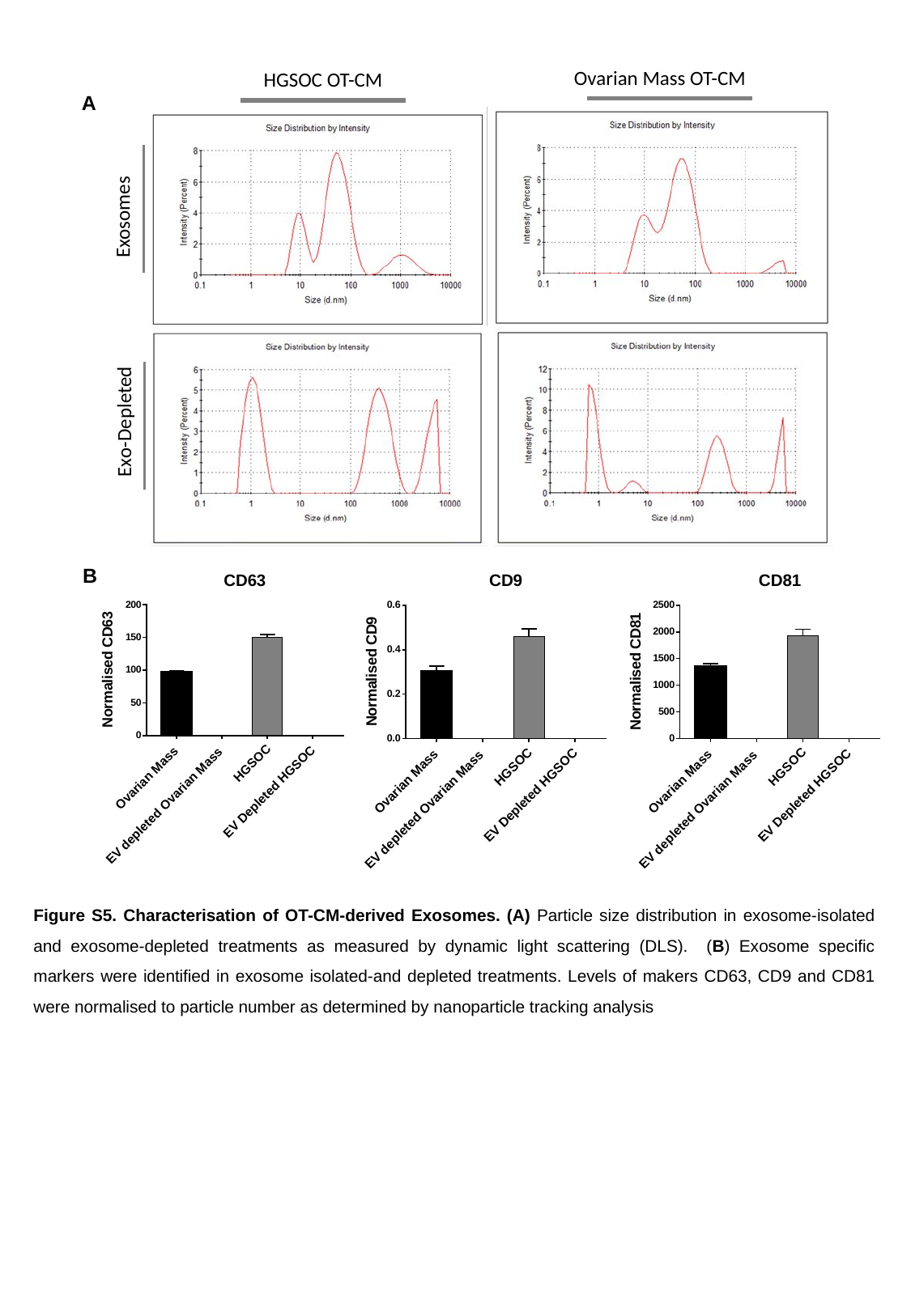

Ovarian Mass OT-CM
HGSOC OT-CM
A
Exosomes
Exo-Depleted
B
Figure S5. Characterisation of OT-CM-derived Exosomes. (A) Particle size distribution in exosome-isolated and exosome-depleted treatments as measured by dynamic light scattering (DLS). (B) Exosome specific markers were identified in exosome isolated-and depleted treatments. Levels of makers CD63, CD9 and CD81 were normalised to particle number as determined by nanoparticle tracking analysis

## Slide 6
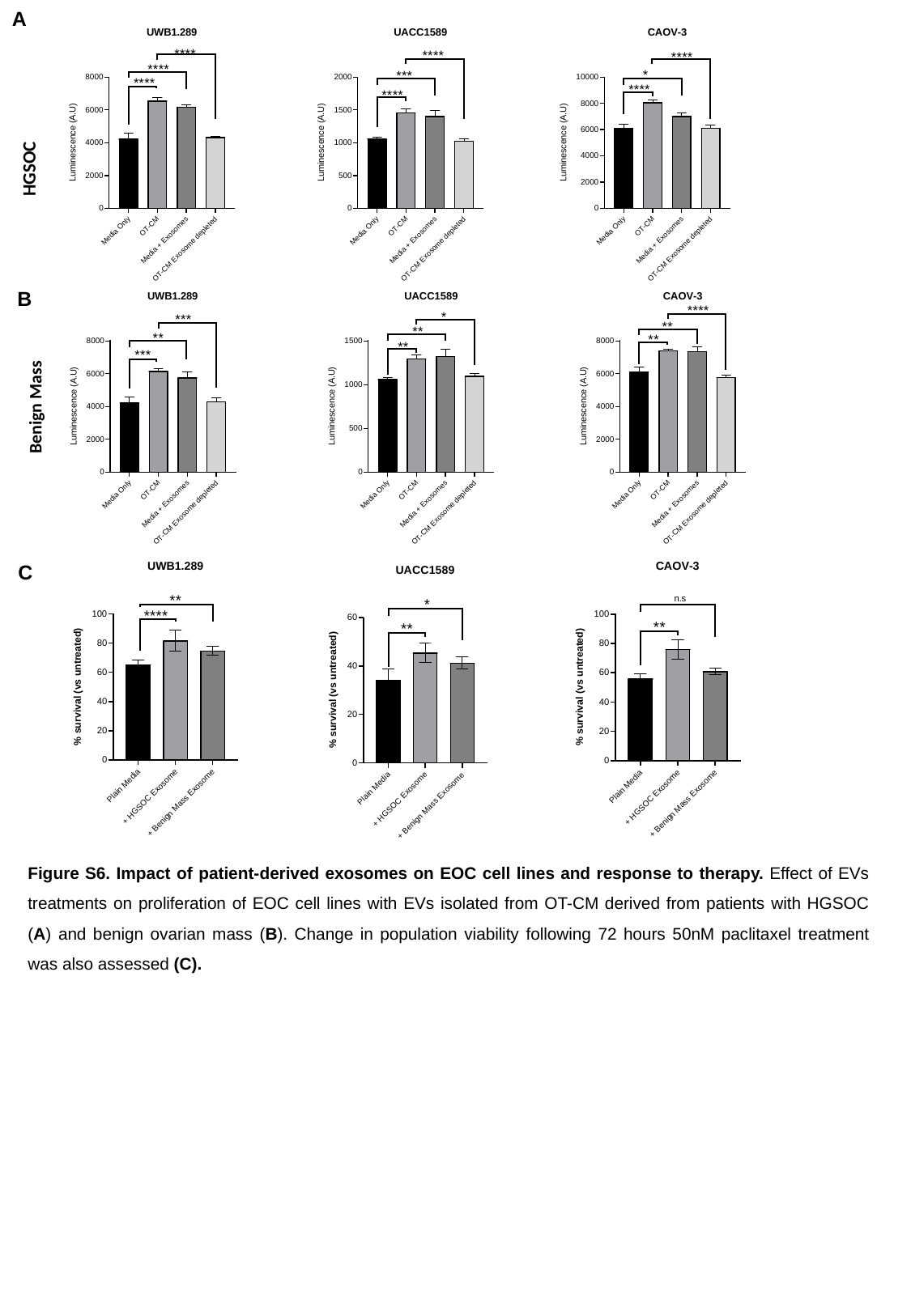

A
HGSOC
B
Benign Mass
C
Figure S6. Impact of patient-derived exosomes on EOC cell lines and response to therapy. Effect of EVs treatments on proliferation of EOC cell lines with EVs isolated from OT-CM derived from patients with HGSOC (A) and benign ovarian mass (B). Change in population viability following 72 hours 50nM paclitaxel treatment was also assessed (C).
