# Supplementary material for: Adipocyte derived exosomes promote cell invasion and challenge paclitaxel efficacy in ovarian cancer
Source: Cell Commun Signal. 2024 Sep 16;22:443. doi: 10.1186/s12964-024-01806-4 (PMC11404028; doi:10.1186/s12964-024-01806-4)
Supplement: Supplementary file 2 — Supplementary Material 2 [file 12964_2024_1806_MOESM2_ESM.pptx]

## Slide 1
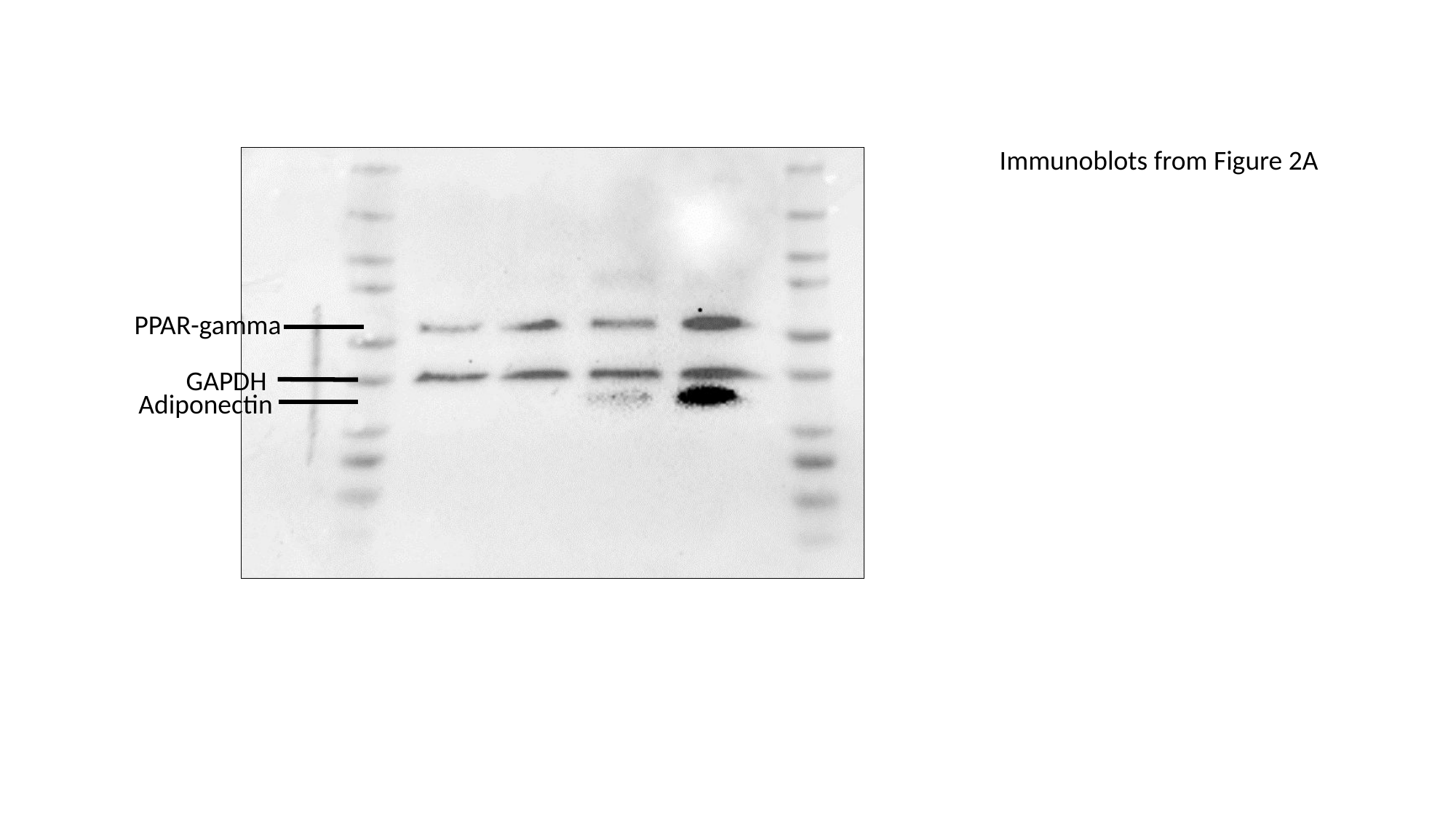

Immunoblots from Figure 2A
PPAR-gamma
GAPDH
Adiponectin

## Slide 2
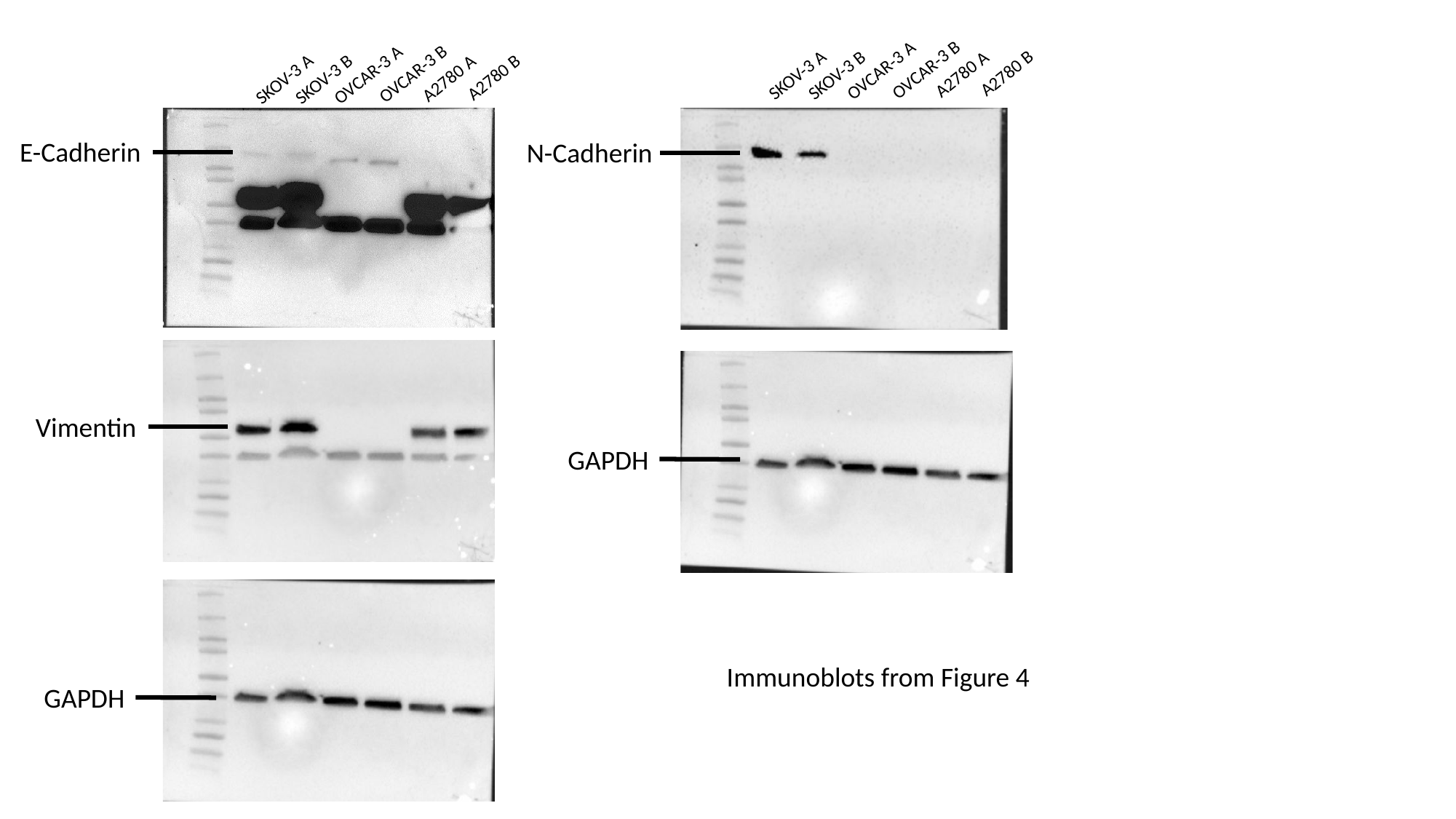

OVCAR-3 B
OVCAR-3 A
A2780 B
OVCAR-3 B
A2780 A
OVCAR-3 A
SKOV-3 A
SKOV-3 B
A2780 B
A2780 A
SKOV-3 A
SKOV-3 B
E-Cadherin
N-Cadherin
Vimentin
GAPDH
Immunoblots from Figure 4
GAPDH

## Slide 3
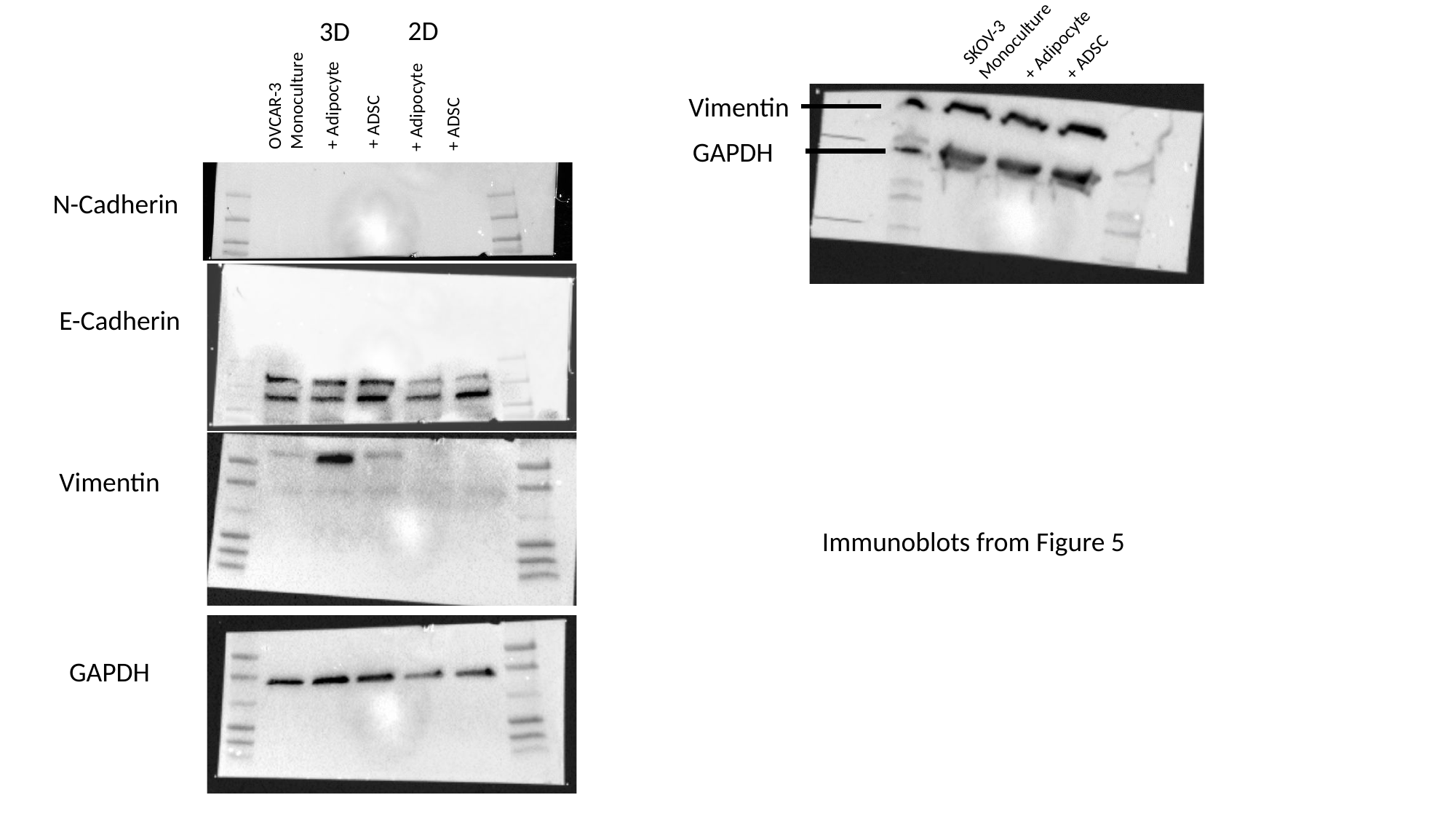

SKOV-3
Monoculture
2D
3D
+ Adipocyte
+ ADSC
OVCAR-3
Monoculture
Vimentin
+ Adipocyte
+ Adipocyte
+ ADSC
+ ADSC
GAPDH
N-Cadherin
E-Cadherin
Vimentin
Immunoblots from Figure 5
GAPDH

## Slide 4
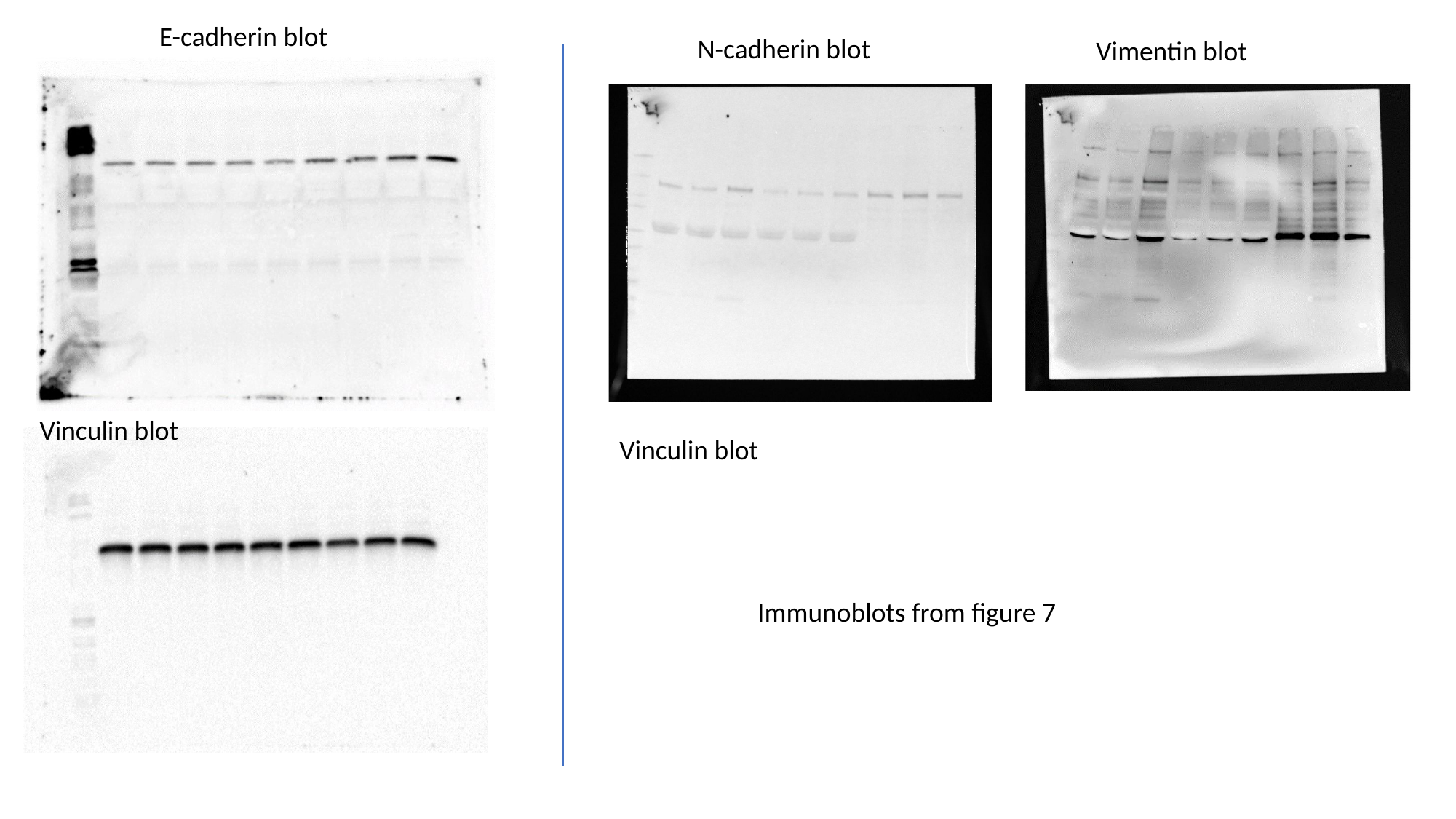

E-cadherin blot
N-cadherin blot
Vimentin blot
Vinculin blot
Vinculin blot
Immunoblots from figure 7
